# Paradoxical evidence weighting in confidence judgments for detection and discrimination

**DOI:** 10.3758/s13414-023-02710-8

**Published:** 2023-06-20

**Authors:** Matan Mazor, Roni O. Maimon-Mor, Lucie Charles, Stephen M. Fleming

**Affiliations:** 1 Department of Psychological Sciences, Birkbeck, University of London, London, UK; 2 Wellcome Centre for Human Neuroimaging, University College London, London, UK; 3 Department of Experimental Psychology, University College London, London, UK; 4 UCL Institute of Ophthalmology, University College London, London, UK; 5 School of Biological and Behavioural Sciences, Queen Mary University of London, London, UK; 6 Institute of Cognitive Neuroscience, University College London, London, UK; 7 Max Planck UCL Centre for Computational Psychiatry and Aging Research, London, UK

**Keywords:** Confidence, Detection, Metacognition

## Abstract

When making discrimination decisions between two stimulus categories, subjective confidence judgments are more positively affected by evidence in support of a decision than negatively affected by evidence against it. Recent theoretical proposals suggest that this “positive evidence bias” may be due to observers adopting a detection-like strategy when rating their confidence—one that has functional benefits for metacognition in real-world settings where detectability and discriminability often go hand in hand. However, it is unknown whether, or how, this evidence-weighting asymmetry affects detection decisions about the presence or absence of a stimulus. In four experiments, we first successfully replicate a positive evidence bias in discrimination confidence. We then show that detection decisions and confidence ratings paradoxically suffer from an opposite “negative evidence bias” to negatively weigh evidence even when it is optimal to assign it a positive weight. We show that the two effects are uncorrelated and discuss our findings in relation to models that account for a positive evidence bias as emerging from a confidence-specific heuristic, and alternative models where decision and confidence are generated by the same, Bayes-rational process.

When considering two alternative hypotheses, the probability of the chosen hypothesis being correct is a function of the availability of evidence supporting not only the chosen hypothesis but also the unchosen one. For example, when deciding that there are more ants in the kitchen than in the living room, confidence should not only positively weigh the number of ants found in the kitchen (*positive evidence*) but also negatively weigh the number of ants found in the living room (*negative evidence*). Specifically, a decision should be based on the difference in the number of ants between the kitchen and the living room, but not on the total number of ants found in both rooms together (we refer to these quantities as *relative evidence* and *sum evidence*, respectively).

While sum evidence is irrelevant to discrimination decisions between two symmetrical hypotheses (e.g., kitchen or living room), it is highly informative with respect to detection decisions about the presence or absence of a signal. For example, when deciding that an ant colony is nesting in the house, we should also care about the total number of ants, irrespective whether they are found in the kitchen or living room (see Fig. 1).

**Fig. 1:**
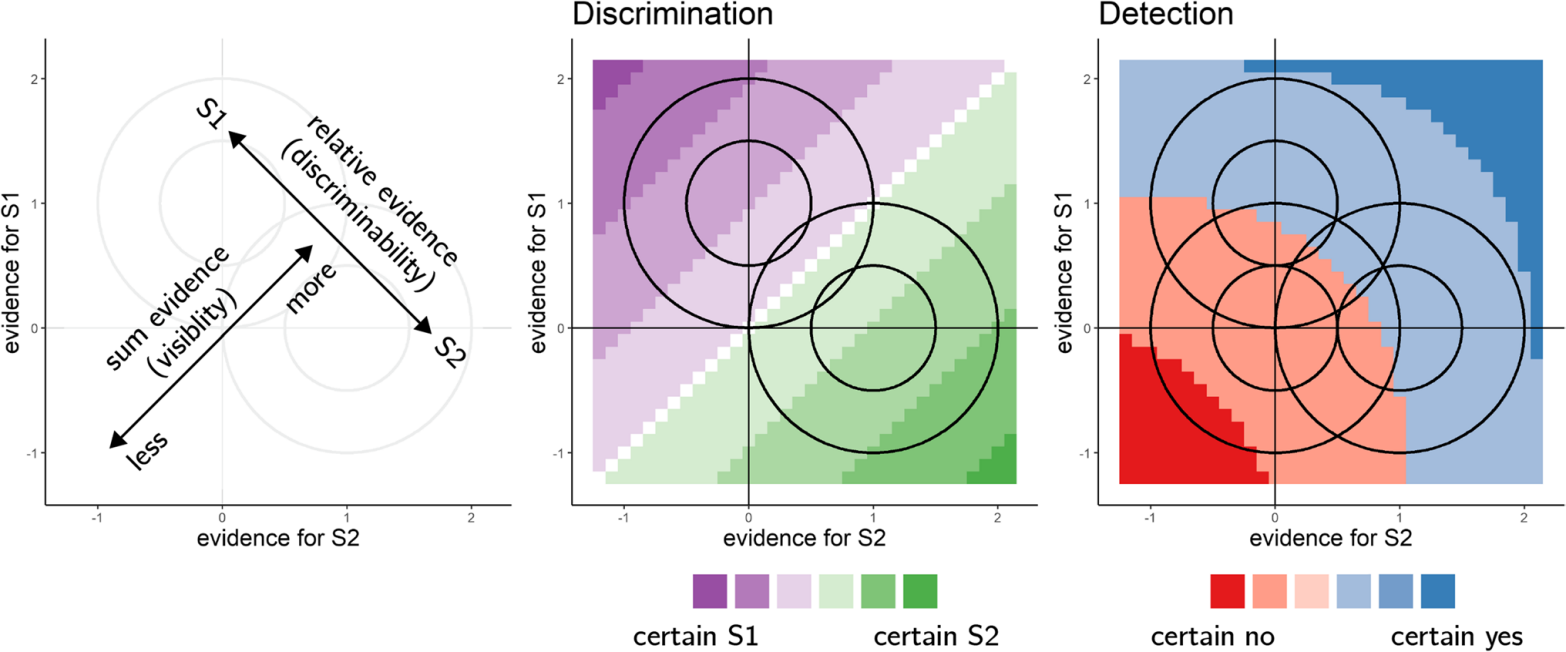
Discrimination and detection in a two-dimensional signal detection theory (SDT) model. Left: In a two-dimensional SDT model, evidence *e* is sampled from one of two Gaussian distributions (here, centered at [0,1] and [1,0]). We define relative evidence as *e*_*S*1_ − *e*_*S*2_ and sum evidence as *e*_*S*1_ + *e*_*S*2_. Circles represent contours of two-dimensional distributions. Center and Right: Decision and confidence accuracy are maximized when based on a log-likelihood ratio for the two stimulus categories. Center: In discrimination, this yields optimal decision and confidence criteria that are based on relative evidence (distance from the main diagonal), irrespective of sum evidence. Right: In detection, this yields optimal decision and confidence that are based on a nonlinear interaction between relative and sum evidence. The third circle centred at (0,0) represents the two-dimensional distribution of percepts in the absence of stimuli. (Colour figure online)

A surprising finding is that, despite the irrelevance of sum evidence to the accuracy of discrimination decisions, people are systematically more confident in their perceptual discrimination decisions when sum evidence is high. For example, Zylberberg et al. (2012) had subjects judge which of two flickering stimuli was brighter on average. Subjects were more confident in their decisions when both stimuli were brighter, indicating an effect of sum evidence (here, overall luminance) on decision confidence. A positive effect of sum evidence on decision confidence is mathematically equivalent to a disproportional weighting of positive evidence over negative evidence, also known as a positive evidence bias (Koizumi et al., 2015; Peters et al., 2017; Rollwage et al., 2020; Samaha & Denison, 2020; Sepulveda et al., 2020; Zylberberg et al., 2012). The two are equivalent because positively weighing the sum of positive and negative evidence effectively weakens the negative contribution of negative evidence to decision confidence, while strengthening the contribution of positive evidence. Notably, this finding stands in contrast to what is expected from the exponential scaling of sensory noise relative to stimulus energy (Weber’s law). Instead, an effect of sum evidence on discrimination confidence may indicate a profound link between how confidence is formed in general, and the processes underpinning perceptual detection (Rausch et al., 2018; Samaha et al., 2020).

Different models identify the origin of this evidence-weighting asymmetry at different levels of the cognitive hierarchy, ranging from positing a metacognitive bias that ignores conflicting information (Maniscalco et al., 2016; metacognitive level, Peters et al., 2017), to asymmetries in the active sampling of evidence (attention allocation level; Sepulveda et al., 2020), and down to perceptual asymmetries between the representations of signal and noise (perception level; Miyoshi & Lau, 2020; Webb et al., 2021). These models vary in whether they postulate separate evidence accumulation processes for decisions and confidence judgments, and in whether they model confidence formation as following a suboptimal heuristic, or alternatively as being optimal with respect to available information (information which may be limited or corrupted by noise).

Here we focus on a subset of models which assume that subjects are rational decision makers equipped with veridical beliefs about the world, but who only have limited access to noisy evidence. Our models further assume that subjects’ confidence ratings are Bayesian estimates of the probability of being correct, given the exact same evidence that was used to make the decision. The models do not postulate any metacognitive biases, heuristics, or suboptimalities. We show that two of these models reproduce a positive evidence bias (that is, a positive effect of sum evidence) in discrimination confidence. The same models also make predictions for evidence weighting in detection judgments and confidence ratings. In four experiments, reverse correlation analysis revealed evidence weighting patterns that only partly agree with the predictions of our models. Most notably, our four models fail to account for a negative evidence bias we observed in detection decisions and confidence: a tendency to irrationally place a negative weighting on evidence, such as being more confident in the presence of a bright stimulus when one of the presented stimuli was unusually dark. In what follows we first describe the four models and the predictions they make, before turning to empirical findings from our four experiments.

## Computational models

**Fig. 2:**
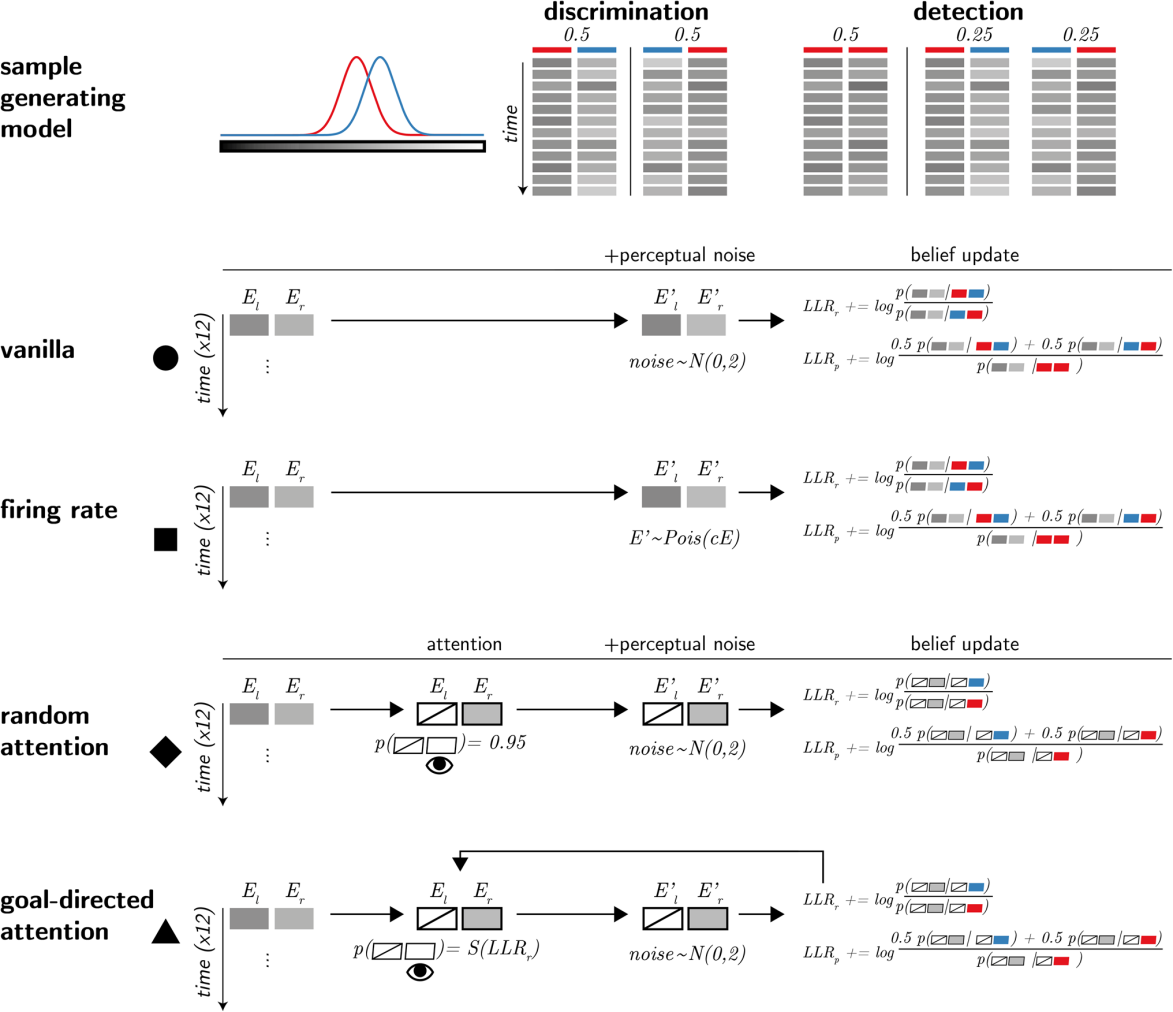
Computational models. Upper panel: True world model. Stimuli span 12 time points, each comprising values from two sensory channels (here, presented as luminance values). In discrimination blocks, values in one channel are sampled from the noise distribution (red), and values in the other channel are sampled from the signal distribution (blue). In detection blocks, on half of the trials, all values are sampled from the noise distribution (red). Vanilla model: On each time point, participants perceive both channels, corrupted by sensory noise that is sampled from a normal distribution. They then update their beliefs accordingly. Firing rate model: Sensory samples are sampled from a Poisson distribution. Random attention model: Agents only attend one channel at a time. The attended channel is chosen at random per time point, with a strong bias which is consistent within a trial. Goal-directed attention model: Channels that are likely to include signal (as determined by previous samples) are more likely to be attended. (Colour figure online)

We model a setting in which agents are presented with a sequence of samples from two noisy sensory channels: *E*_1_ and *E*_2_. The agents’ task is to decide which of the two channels was the signal channel (discrimination), or whether any of the channels had signal in it at all (detection). When a signal is present in a channel, evidence *E* is sampled from a normal distribution $\mathcal{N}\left(0.5,1\right)$, and when a signal is absent evidence is sampled from $\mathcal{N}\left(0,1\right)$ (see Fig. 2, upper panel). In all four models agents only have access to a noisy version of these samples *E*^′^, corrupted by additional internal sensory noise. After each time step, they update their belief about the relative likelihood of the observed samples under the two possible world states (signal in Channel 1 versus 2, or signal presence versus absence), and given full knowledge of the true sample-generating process, including the properties of sensory noise. Each trial comprises 12 time steps. At the end of a trial, agents report the world state that maximizes the likelihood of the observed evidence, and rate their confidence as the objective probability that their decision was correct given the accumulated likelihood estimates. The four models vary in the properties of sensory noise, and in the selection of some channels for inspection by selection mechanisms.

### Vanilla model

In the basic, vanilla model, sensory noise is sampled from a normal distribution $\mathcal{N}\left(0,2\right)$. This model corresponds to a standard equal-variance signal detection model, as illustrated in Fig. 1.

### Firing rate model

The firing rate model is similar to the vanilla model, with the exception that perceived values are sampled from a Poisson, rather than a normal distribution. An important property of the Poisson distribution family, commonly used to model firing rates in neuronal populations, is that their mean and variance are lawfully coupled: the stronger the activation, the more variable it is. When applied to sensory neurons, this results in strong stimuli being subjectively perceived as noisier, consistent with the Weber–Fechner law (Fechner & Adler, 1860). In identifying the origin of the positive evidence bias at the perceptual level, this model shares a family resemblance with the unequal-variance model by Miyoshi and Lau (2020). An important feature of this model is that perceptual noise is conditioned not on stimulus class, but on the perceptual sample. This seems plausible, as the perceptual system has no access to stimulus class beyond the information that is available in perceptual samples.

### Random attention model

Like the vanilla model, sensory noise is again sampled from $\mathcal{N}\left(0,2\right)$. Unlike the vanilla model, however, here agents have access to one channel per time point only (they ‘attend’ to one channel at a time). At the start of each trial, agents randomly choose a preferred channel. Then, on each time point, they attend to the preferred channel with probability 0.95, and the nonpreferred channel with probability 0.05, and update their beliefs accordingly. We include this model because it is inherently asymmetric: on each trial, evidence from the preferred channel contributes more to both decision and confidence, simply because it is more visible to the agent.

### Goal-directed attention model

This model is similar to the random attention model, except that here attention is biased towards channels that are more likely to include signal. Specifically, agents track the log likelihood ratio *LLR*_*r*_ between signal presence in the left or in the right channels, with the probability of attending the right channel being dynamically set at each time point to *S*(*LLR*_*r*_) where *S* is a sigmoid function with a steep slope of 5 and *LLR*_*r*_ is based on all previous sensory samples in the trial. A conceptually similar drift diffusion model was previously shown to produce a positive evidence bias in confidence ratings (Sepulveda et al., 2020).

## Simulations

We simulated 20,000 discrimination and 20,000 detection trials per model (100 trials × 200 simulated agents per model). On each discrimination trial, the signal channel was designated as right or left with equal probability. On half of the detection trials both channels were noise channels. We then sampled, for each trial, 12 values from each channel. These 24 values were then passed on to the simulated agent, who returned a decision and a confidence rating. We then subjected the agents’ decisions and confidence ratings to a reverse correlation analysis. We now turn to describe this analysis, which will also be used to analyze the behaviour of human participants in Exps. 1–4.

### Reverse correlation analysis

Following Zylberberg et al. (2012), we took a reverse correlation approach and asked which sources of evidence (positive, negative, relative, and sum evidence) contribute to agents’ decisions and confidence ratings. This analysis focuses on random fluctuations in signal intensity, and asks how they affect behaviour (here, decisions and confidence in these decisions). Accordingly, in analyzing data from our simulated agents, we contrasted external stimulus energy (*E*) and not internal stimulus energy (*E*^’^) leaving internal noise hidden.

#### Methodological note: Positive evidence bias in perceptual decisions

The positive evidence bias in decision confidence is often seen as particularly striking, given that positive and negative evidence are equally weighted in forming a decision (Peters et al., 2017; Zylberberg et al., 2012). For example, using reverse correlation, Zylberberg et al. (2012) showed that momentary fluctuations in the availability of perceptual evidence for and against a decision were equally predictive of the decision itself. Similarly, Peters et al. (2017) showed that in classifying rapidly presented images as ‘face’ or ‘house’, decisions are not solely guided by positive evidence (e.g., face-related brain activity when deciding ‘face’), but also by negative evidence (e.g., house-related brain activity when deciding ‘face’).

In both cases, it is useful to ask what it would look like for an agent to only consider positive evidence in making a decision. This soon becomes circular, because positive and negative evidence are defined with respect to the decision itself. For example, when analyzing the decisions of an agent that consistently ignores evidence for one alternative (similar to the random attention model above), both positive and negative evidence should still be predictive of decisions. The effect of positive evidence is then driven by those trials in which the agent selected the attended alternative, and the effect of negative evidence by those trials in which the agent selected the ignored alternative (because the evidence for the attended alternative was insufficient). Put differently, asymmetries of positive and negative evidence cannot affect the decision itself, because at the time of making the decision there is no positive and negative evidence to speak of—instead, there are two sources of evidence that may become positive or negative, depending on the decision that is selected. For this reason, in measuring evidence weighting in decision formation, we defined relative and sum evidence relative to the ground truth rather than the agents’ decision.

#### Discrimination decisions

From each trial (*tr*) we extracted random fluctuations in perceptual evidence in the signal ${E}_s^{tr}(t)$ and nonsignal ${E}_n^{tr}(t)$ sensory channels. To make sure we are measuring true random fluctuations and not systematic differences between noise and signal channels, we mean centered the signal channels across trials to 0, such that the average time course across all agents and trials was constant at 0. For simplicity, in extracting qualitative predictions from model simulations we averaged all time points in a trial to obtain trial-level estimates ${E}_s^{tr}$ and ${E}_n^{tr}$. Human data were analyzed in a similar fashion, but separately for each time point. Time-resolved decision and confidence kernels derived from model simulations are available in the Appendix.

**Fig. 3:**
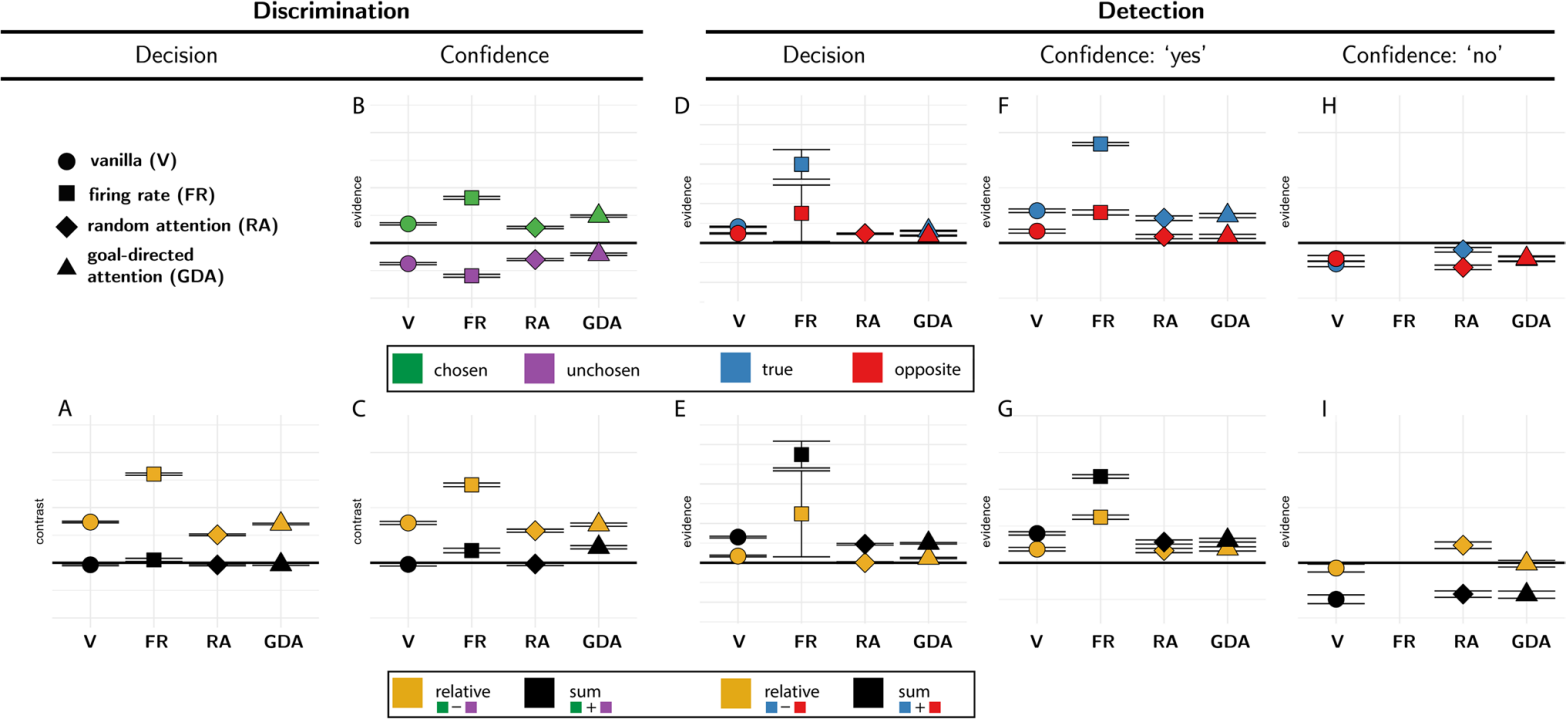
Simulated predictions for the reverse correlation analysis, derived from the four models. **A** Effects of relative (orange markers) and sum (black markers) evidence on discrimination decisions. **B** Effects of evidence for the chosen (green markers) and unchosen (purple markers) alternatives on discrimination confidence. **C** Effects of sum and relative evidence (defined with respect to participants’ decisions) on discrimination confidence. **D, F,** and **H** Effects of evidence in the signal channel (blue markers) and in the nonsignal channel (red markers) on detection decisions, confidence in yes responses, and confidence in no responses, respectively. **E, G,** and **I** Effects of relative evidence (orange markers) and sum evidence (black markers) on detection decisions, confidence in yes responses, and confidence in no responses, respectively. For scale, grid lines are plotted in common arbitrary units

‘Relative evidence’ was defined as the difference in noise terms between the signal and nonsignal channels (${E}_{relative}^{tr}={E}_s^{tr}-{E}_n^{tr}$). To obtain a decision kernel, we took the difference between the average relative evidence in trials where agents chose the signal and nonsignal channels ${E}_{relative}={\left\langle {E}_{relative}^{tr}\right\rangle}_{CORRECT}-{\left\langle {E}_{relative}^{tr}\right\rangle}_{INCORRECT}$. This was done separately for each simulated agent, and the resulting values were tested against zero in a *t* test. In all four models, relative evidence was higher on trials in which the agent correctly identified the signal channel (Fig. 3A, orange markers).

‘Sum evidence’ was defined as the total sum of noise terms across both channels (${E}_{sum}^{tr}={E}_s^{tr}+{E}_n^{tr}$). Similarly, we used the difference between sum evidence in correct and incorrect trials ${E}_{sum}={\left\langle {E}_{sum}^{tr}\right\rangle}_{CORRECT}-{\left\langle {E}_{sum}^{tr}\right\rangle}_{INCORRECT}$ to probe effects of sum evidence on decision. Sum evidence had no effect on decision in any of the four models (Fig. 3A, black markers).

#### Discrimination confidence

In all four models, confidence was defined as the Bayesian probability of being correct, given an equal prior over the two world states (see Appendix). The median confidence rating was used to split evidence channels into four sets, according to decision (chosen or unchosen, depending on the agent’s decision) and confidence level (high or low). Confidence kernels for the chosen and unchosen channels were then extracted by subtracting the mean low-confidence from the mean high-confidence values for each channel:

$${E}_{conf- chosen}={\left\langle {E}_{chosen}^{tr}\right\rangle}_{HIGH}-{\left\langle {E}_{chosen}^{tr}\right\rangle}_{LOW}$$

$${E}_{conf- unchosen}={\left\langle {E}_{unchosen}^{tr}\right\rangle}_{HIGH}-{\left\langle {E}_{unchosen}^{tr}\right\rangle}_{LOW}$$

Confidence kernels were also extracted for relative and sum evidence:

$${E}_{conf- relative}=\left({\left\langle {E}_{chosen}^{tr}\right\rangle}_{HIGH}-{\left\langle {E}_{unchosen}^{tr}\right\rangle}_{HIGH}\right)-\left({\left\langle {E}_{chosen}^{tr}\right\rangle}_{LOW}-{\left\langle {E}_{unchosen}^{tr}\right\rangle}_{LOW}\right)$$

$${E}_{conf- sum}=\left({\left\langle {E}_{chosen}^{tr}\right\rangle}_{HIGH}+{\left\langle {E}_{unchosen}^{tr}\right\rangle}_{HIGH}\right)-\left({\left\langle {E}_{chosen}^{tr}\right\rangle}_{LOW}+{\left\langle {E}_{unchosen}^{tr}\right\rangle}_{LOW}\right)$$

In all four models, high confidence ratings were associated with stronger evidence in the chosen channel (Fig. 3B, green markers) and weaker evidence in the unchosen channel (Fig. 3B, purple markers). As expected, this translated to an effect of relative evidence on decision confidence: agents were more confident when the evidence difference between the chosen and unchosen channels (*E*_*conf* − *relative*_) was high (Fig. 3C, orange markers).

Critically, only the firing rate and goal-directed attention models produced an effect of sum evidence (*E*_*conf* − *sum*_) on decision confidence, such that agents were more confident when overall evidence was high (Fig. 3C, black markers). As reviewed above, this effect is consistent with a positive evidence bias in discrimination confidence.

#### Detection decisions

For the reverse correlation analysis of detection decisions, we focused on trials in which a signal was present. This allowed us to disentangle the effects of evidence in the signal and nonsignal channels on detection decisions and confidence. We subtracted evidence in trials that resulted in a ‘no’ (target absent) decision from evidence in trials that resulted in a ‘yes’ (target present) decision, separately for the signal and nonsignal channels:

$${E}_{detection-s}={\left\langle {E}_s^{tr}\right\rangle}_{YES}-{\left\langle {E}_s^{tr}\right\rangle}_{NO}$$

$${E}_{detection-n}={\left\langle {E}_n^{tr}\right\rangle}_{YES}-{\left\langle {E}_n^{tr}\right\rangle}_{NO}$$

We similarly obtained detection kernels as a function of relative and sum evidence:

$${E}_{detection- relative}=\left({\left\langle {E}_s^{tr}\right\rangle}_{YES}-{\left\langle {E}_n^{tr}\right\rangle}_{YES}\right)-\left({\left\langle {E}_s^{tr}\right\rangle}_{NO}-{\left\langle {E}_n^{tr}\right\rangle}_{NO}\right)$$

$${E}_{detection- sum}=\left({\left\langle {E}_s^{tr}\right\rangle}_{YES}+{\left\langle {E}_n^{tr}\right\rangle}_{YES}\right)-\left({\left\langle {E}_s^{tr}\right\rangle}_{NO}+{\left\langle {E}_n^{tr}\right\rangle}_{NO}\right)$$

In all four models, ‘yes’ responses were associated with stronger evidence in the signal channel (Fig. 3D, blue markers). Importantly, the same was true for evidence in the nonsignal channel: Agents were more likely to respond ‘yes’ when evidence was stronger in this channel too (Fig. 3D, red markers). This is a key prediction of our Bayes-rational models: In detection, evidence in both channels should be weighted positively, as the agent’s goal is to detect any signal relative to noise. Together, these two positive effects translated to a strong effect of sum evidence on detection decisions: Agents were more likely to respond ‘yes’ when the total sum of evidence was high (Fig. 3E, black markers). A weaker effect of relative evidence on detection decisions was observed in all models except for the random attention model (Fig. 3E, orange markers).

#### Detection confidence

Similar to the discrimination task, the median confidence rating was used to split evidence channels into four sets, according to signal (signal channel or nonsignal channel) and confidence level (high or low). This was done separately for ‘yes’ and ‘no’ responses. Confidence kernels for the signal and nonsignal channels were then extracted by subtracting the mean low-confidence from the mean high-confidence evidence values for each channel and decision. For example, for ‘yes’ responses this meant computing:

$${E}_{conf- yes-s}={\left\langle {E}_s^{tr}\right\rangle}_{YES, HIGH}-{\left\langle {E}_s^{tr}\right\rangle}_{YES, LOW}$$

$${E}_{conf- yes-n}={\left\langle {E}_n^{tr}\right\rangle}_{YES, HIGH}-{\left\langle {E}_n^{tr}\right\rangle}_{YES, LOW}$$

$${E}_{conf- yes- relative}=\left({\left\langle {E}_s^{tr}\right\rangle}_{YES, HIGH}-{\left\langle {E}_n^{tr}\right\rangle}_{YES, HIGH}\right)-\left({\left\langle {E}_s^{tr}\right\rangle}_{YES, LOW}-{\left\langle {E}_n^{tr}\right\rangle}_{YES, LOW}\right)$$

$${E}_{conf- yes- sum}=\left({\left\langle {E}_s^{tr}\right\rangle}_{YES, HIGH}+{\left\langle {E}_n^{tr}\right\rangle}_{YES, HIGH}\right)-\left({\left\langle {E}_s^{tr}\right\rangle}_{YES, LOW}+{\left\langle {E}_n^{tr}\right\rangle}_{YES, LOW}\right)$$

In all four models, agents were more confident in their decisions about signal presence when evidence in the signal channel was stronger (Fig. 3F, blue markers). Mirroring the detection decision kernel means, confidence in signal presence was also positively affected by evidence for signal in the nonsignal channel (Fig. 3F, red markers). Together, these two positive effects produced an overall positive effect of sum evidence on confidence in signal presence (Fig. 3G, black markers). All four models predicted a weaker effect of relative evidence (Fig. 3G, orange markers).

Finally, we asked how random variability in sensory noise contributed to confidence in detection “no” responses. Here, a low number of misses made it difficult to reliably estimate confidence kernels for the firing rate model. In the remaining three models, agents were more confident in decisions about signal absence when evidence in both signal and nonsignal channels was weaker (Fig. 3H, blue and red markers, respectively). Together, these negative effects translated to a total negative effect of sum evidence on confidence in absence (Fig. 3I, black markers). None of the four models predicted a negative effect of relative evidence on confidence in absence, but the random attention model predicted a subtle positive effect (Fig. 3I, orange markers).

Equipped with qualitative predictions from four Bayes-rational models, we now turn to describing our empirical results. As we report below, these models failed to account for a key signature of human decision making: in both decisions and confidence ratings, subjects negatively weigh evidence in the nonsignal channel when inferring signal presence, as if they are making a discrimination judgment about the origin of the signal, rather inferring signal presence.

## Experiment 1

### Methods

#### Participants

The research complied with all relevant ethical regulations and was approved by the Research Ethics Committee of University College London (UCL; study ID number 1260/003). Ten participants were recruited via the UCL’s psychology subject pool, and gave their informed consent prior to their participation. Each participant performed four sessions of 600 trials each, in blocks of 100 trials. Sessions took place on different days and consisted of three discrimination blocks interleaved with three detection blocks.

#### Experimental procedure

The experimental procedure for Exp. 1 largely followed the procedure described in Zylberberg et al. (2012), Exp. 1. Participants observed a random-dot kinematogram for a fixed duration of 700 ms. In discrimination trials, the direction of motion was one of two opposite directions with equal probability, and participants reported the observed direction by pressing one of two arrow keys on a standard keyboard. In detection blocks, participants reported whether there was any coherent motion by pressing one of two arrow keys on a standard keyboard. In half of the detection trials, dots moved coherently to one of two opposite directions, and in the other half all dots moved randomly.

**Fig. 4:**
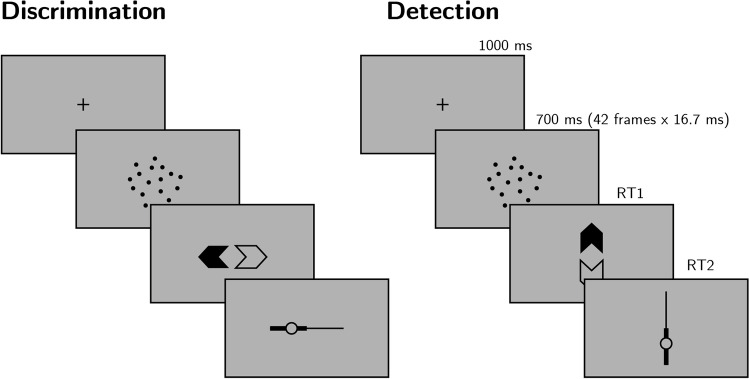
Task design for Experiment 1. In both discrimination and detection blocks, participants viewed 700 ms of a random dot motion array, after which they made a keyboard response to indicate their decision (motion direction in discrimination, signal absence or presence in detection), followed by a continuous confidence report using the mouse. Five participants viewed vertically moving dots and indicated their detection responses on a horizontal scale, and five participants viewed horizontally moving dots and indicated their detection responses on a vertical scale

In both detection and discrimination blocks, participants indicated their confidence following each decision. Confidence was reported on a continuous scale ranging from chance to complete certainty. To avoid systematic response biases affecting confidence reports, the orientation (vertical or horizontal) and polarity (e.g., right or left) of the scale was set to agree with the Type 1 response. For example, following an up-arrow press, a vertical confidence bar was presented where ‘guess’ is at the center of the screen and ‘certain’ appeared at the upper end of the scale (see Fig. 4).

To control for response requirements, for five subjects, the dots moved to the right or to the left, and for the other five subjects, they moved upward or downward. The first group made discrimination judgments with the right and left keys and detection judgments with the up and down keys, and this mapping was reversed for the second group. The number of coherently moving dots (‘motion coherence’) was adjusted to maintain performance at around 70% accuracy for detection and discrimination tasks independently. This was achieved by measuring mean accuracy after every 20 trials, and adjusting coherence by a step of 3% if accuracy fell below 60% or went above 80%. We opted for a block-wise staircasing procedure in order to keep motion energy relatively stable across trials, allowing participants to optimally place their detection criterion. The staircasing procedure for both tasks started at a coherence value of 1.0.

Stimuli for discrimination blocks were generated using the exact same procedure reported in Zylberberg et al. (2012).^1^ Trials started with a presentation of a fixation cross for 1 second, immediately followed by stimulus presentation. The stimulus consisted of 152 white dots (diameter = 0.14^∘^), presented within a 6.5^∘^ circular aperture centered on the fixation point for 700 ms (42 frames, frame rate = 60 Hz). Dots were grouped in two sets of 76 dots each. Every other frame, the dots of one set were replaced with a new set of randomly positioned dots. For each coherence value of *c*^′^, a proportion of *c*^′^ of the dots from the second set moved coherently in one direction by a fixed distance of 0.33^∘^, while the remaining dots in the set moved in random directions by a fixed distance of 0.33^∘^. On the next update, the sets were switched, to prevent participants from tracing the position of specific dots. Frame-specific coherence values were sampled for each screen update from a normal distribution centred around the coherence value *c* with a standard deviation of 0.07, with the constraint that *c*^′^ must be a number between 0 and 1.

Stimuli for detection blocks were generated using a similar procedure, where on a random half of the trials coherence was set to 0%, without random sampling of coherence values for different frames.

To probe global metacognitive estimates of task performance, at the end of each experimental block (100 trials) participants estimated the number of correct responses they have made. Analysis of these global metacognitive estimates is provided in the Appendix.

### Analysis

Experiment 1 was preregistered (preregistration document is available here: https://osf.io/z2s93/). Our full preregistered analysis is available in the Appendix.

#### Reverse correlation analysis

For the reverse correlation analysis, we followed a procedure similar to the one described in Zylberberg et al. (2012). For each of the four directions (right, left, up and down), we applied two spatiotemporal filters to the frames of the dot motion stimuli as described in previous studies (Adelson & Bergen, 1985; Zylberberg et al., 2012). The outputs of the two filters were squared and summed, resulting in a three-dimensional matrix with motion energy in a specific direction as a function of *x, y,* and time. We then took the mean of this matrix across the *x* and *y* dimensions to obtain an estimate of the overall temporal fluctuations in motion energy in the selected direction. Using this filter, we obtained estimates of temporal fluctuations in the mean and variance of motion energy for upward, downward, leftward and rightward motion within each trial. We refer to these temporal estimates as motion energy vectors, where each such vector consists of 42 entries, one per time point. Additionally, for every time point we extracted the variance along the *x* and *y* dimensions, but given the high correlation between our estimates of mean and variance, we focused our analysis on the mean motion energy.

In order to distill random fluctuations in motion energy from mean differences between stimulus categories, we subtracted the mean motion energy from trial-specific motion energy vectors. The mean motion energy vectors were extracted by averaging the motion energy vectors of all participants, separately for each motion coherence level and motion direction. We chose this approach instead of the linear regression approach used by Zylberberg et al. (2012) in order to be sensitive to the possibility of nonlinear effects of coherence on motion energy.

### Results

#### Decision accuracy

Overall proportion correct was 0.74 in the discrimination and 0.72 in the detection task. Performance in discrimination was significantly higher than in detection *M*_*D*_ = 0.02, 95% CI [0.00, 0.04], *t*(9) = 2.43, *p* = .038. This difference in task performance reflected a slower convergence of the staircasing procedure for the discrimination task during the first session. When discarding all data from the first session and analyzing only data from the last three sessions (1,800 trials per participant), task performance was equated between the two tasks at the group level *M*_*D*_ = 0.00, 95% CI [−0.02, 0.02], *t*(9) =  − 0.05, *p* = .962; BF_10_ = 0.31. In order to avoid confounding differences between discrimination and detection decision and confidence profiles with more general task performance effects, the first session was excluded from all subsequent analyses.

#### Overall properties of response time and confidence distributions

In detection, participants were more likely to respond ‘yes’ than ‘no’ (mean proportion of ‘yes’ responses: *M* = 0.59, 95% CI [0.53, 0.64], *t*(9) = 3.45, *p* = .007). We did not observe a consistent response bias for the discrimination data (mean proportion of ‘rightward’ or ‘upward’ responses: *M* = 0.52, 95% CI [0.47, 0.57], *t*(9) = 1.00, *p* = .344).

Replicating previous studies (Kellij et al., 2021; Mazor et al., 2020, 2021; Meuwese et al., 2014), we find the typical asymmetries between detection ‘yes’ and ‘no’ responses in response time, overall confidence, and the alignment between subjective confidence and objective accuracy (also termed metacognitive sensitivity, measured as the area under the response-conditional Type 2 ROC curve). ‘No’ responses were slower compared with ‘yes’ responses (median difference: 85.37 ms), and accompanied by lower levels of subjective confidence (mean difference of 0.08 on a 0–1 scale). Metacognitive sensitivity was higher for detection ‘yes’ compared with detection ‘no’ responses (mean difference in area under the curve units: 0.11). No difference in response time, confidence, or metacognitive sensitivity was found between the two discrimination responses. For a detailed statistical analysis of these behavioural asymmetries, see Appendix.

#### Reverse correlation

##### Discrimination

Using reverse correlation we quantified the effect of random fluctuations in motion energy on the probability of correctly identifying the true direction of motion, and on the temporal dynamics of decision formation. Importantly, this analysis approach treats leftward and rightward motion energy as two independently represented quantities, assuming that the decision-making module has access to individual spatiotemporal filters, and not only to the difference between them (Adelson & Bergen, 1985; Levinson & Sekuler, 1975; Van Santen & Sperling, 1984). We return to this point in describing the rationale for Exp. 2.

**Fig. 5:**
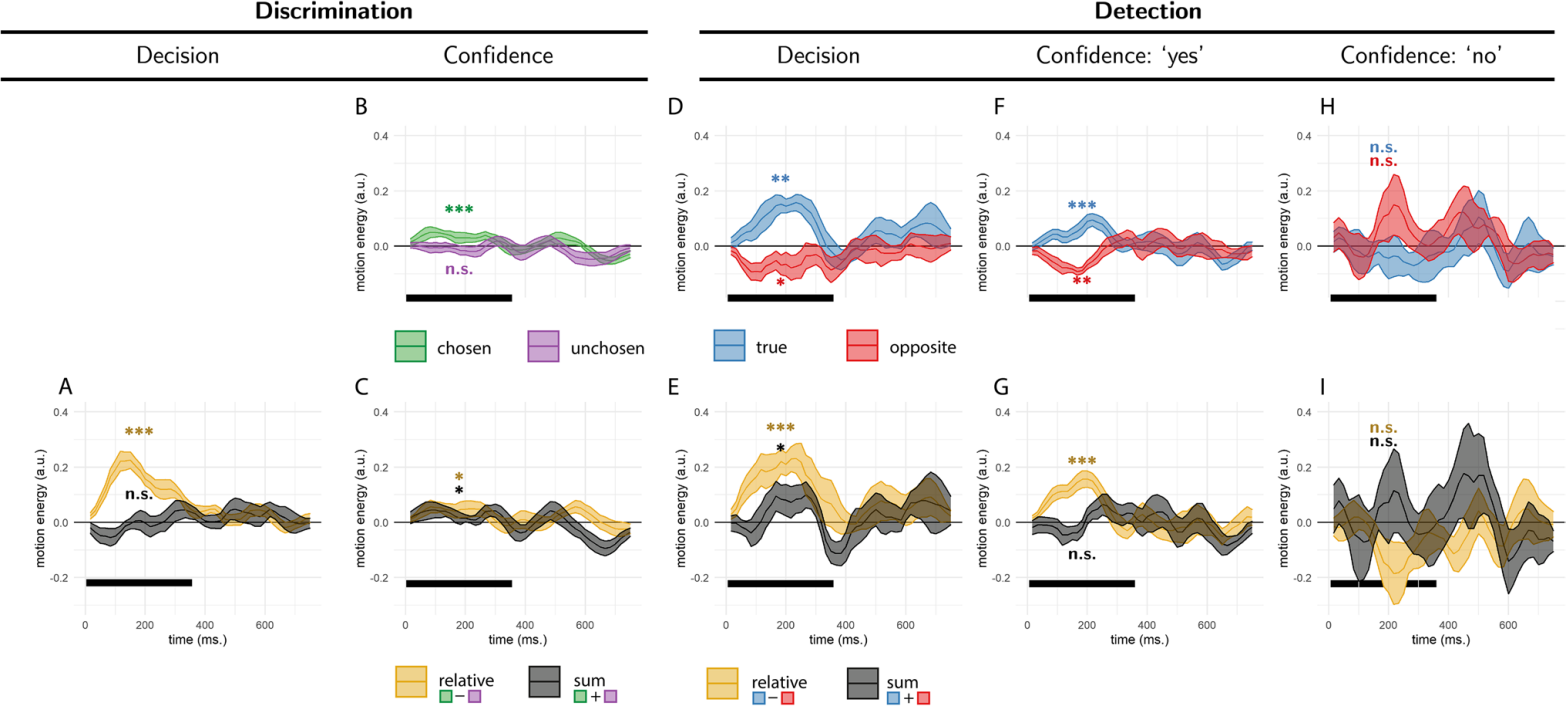
Reverse correlation, Exp. 1. **A** Effects of relative (orange curve) and sum (black curve) evidence on discrimination decisions. Note that relative evidence here is defined with respect to the true direction of motion, not participants’ decisions. **B** Effects of evidence for the chosen (green curve) and unchosen (purple curve) alternative on discrimination confidence. **C** Effects of sum and relative evidence (defined with respect to participants’ decisions) on discrimination confidence. **D, F** and **H** Effects of evidence for the true direction of motion (blue curve) and for the opposite direction of motion (red curve) on detection decisions, confidence in yes responses, and confidence in no responses, respectively. **E, G,** and **I** Effects of relative evidence (orange curve) and sum evidence (black curve) on detection decisions, confidence in yes responses, and confidence in no responses, respectively. The first 300 ms of the trial are marked in black. All nine panels are presented at the same scale, in arbitrary motion-energy units. Stars represent significance in a two-sided *t* test for the first 300 ms of the trial: *p < .05, **p < .01, ***p < .001. (Colour figure online)

Following Zylberberg et al. (2012), we focused our analysis on the first 300 ms of the trial. Participants’ discrimination responses were significantly affected by the relative evidence for the true direction of motion compared with the opposite direction, (*E*_*relative*_; *t*(9) = 8.48, *p* < .001), whereas sum evidence (*E*_*sum*_; the total amount of energy in both directions) had no effect on discrimination accuracy, (*t*(9) =  − 0.70, *p* = .502 see Fig. 5A). This is consistent with a symmetric weighting of evidence in decision formation, and with the predictions of all four models.

We next turned to the contribution of motion energy to subjective confidence ratings. The median confidence rating in each experimental session was used to split all motion energy vectors into four groups, according to decision (chosen or unchosen directions) and confidence level (high or low). Confidence kernels for the chosen and unchosen directions were then extracted by subtracting the mean low-confidence from the mean high-confidence vectors for both the chosen and unchosen directions. Motion energy in the chosen direction (positive evidence) significantly increased confidence (*E*_*conf* − *chosen*_, *t*(9) = 4.99, *p* = .001), but we found no significant decrease in confidence with stronger motion energy in the opposite direction, (*E*_*conf* − *unchosen*,_ *t*(9) =  − 0.25, *p* = .807; see Fig. 5B). Equivalently, both relative and sum evidence positively contributed to decision confidence (*E*_*conf* − *relative*_, *t*(9) = 2.76, *p* = .022; *E*_*conf* − *sum*_: *t*(9) = 2.92, *p* = .017; see Fig. 5C). This is a replication of the positive evidence bias observed in Zylberberg et al. (2012), and consistent with the predictions of the firing rate and goal-directed attention models.

##### Detection

Participants were significantly more likely to respond ‘yes’ when fluctuations in motion energy during the first 300 ms of the trial strengthened motion energy in the true direction of motion (*E*_*detection* − *s*_, *t*(9) = 6.06, *p* < .001; see Fig. 5D, blue curve). Critically, and in contrast to the predictions of all four Bayes-rational models, motion energy in the opposite direction had a negative, rather than a positive effect on the probability of responding ‘yes’ (*E*_*detection* − *n*_, *t*(9) =  − 2.89, *p* = .018; see Fig. 5D, red curve). In other words, stronger motion energy in the opposite direction made it less likely that people would say a signal was present.

Confidence ratings were higher in detection ‘yes’ responses when random noise strengthened the motion energy in the true direction of motion (*E*_*conf* − *yes* − *s*_; *t*(9) = 4.59, *p* = .001; see Fig. 5F, blue curve). Again, in contrast to our model predictions, motion energy in the opposite direction had a negative, rather than a positive effect on detection confidence. That is, subjects were more confident in the presence of coherent motion when there was an unusually low level of motion energy in one of the two directions (*E*_*conf* − *yes* − *n*_; *t*(9) =  − 2.95, *p* = .016; see Fig. 5F, red curve).

Furthermore, unlike in the discrimination task, we found no effect of sum evidence on confidence ratings in ‘yes’ responses (*E*_*conf* − *yes* − *sum*_; *t*(9) = 0.14, *p* = .892; see Fig. 5G, black curve). To reiterate, while detection *decisions* were mostly sensitive to fluctuations in motion energy toward the true direction of motion, *confidence judgments* in detection ‘yes’ responses were equally sensitive (with opposite signs) to fluctuations in the true and opposite directions of motion. However, and to anticipate the results of Exp. 3, presented below, we note that this symmetric weighting of evidence in detection confidence was not replicated in a subsequent experiment designed to directly test this surprising result.

**Fig. 6:**
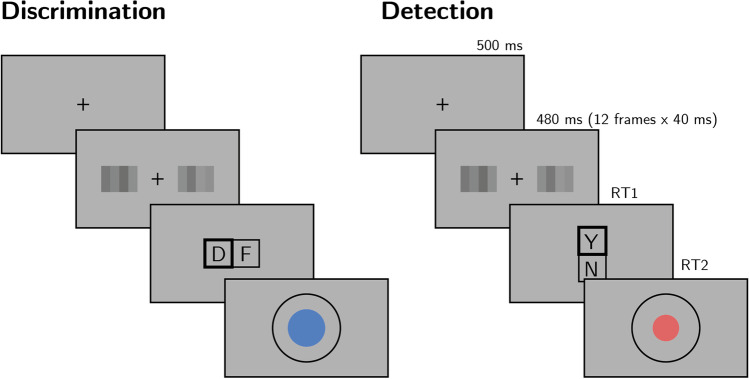
Task design for Experiment 2. In both tasks, participants viewed two flickering patches for 480 ms, after which they made a keyboard response to indicate which of the patches was brighter (discrimination) or whether any of the patches was brighter than the background (detection). (Colour figure online)

Finally, confidence in ‘no’ responses was independent of relative, sum, positive, and negative evidence (all *p*s > 0.1; see Fig. 6H).

## Experiment 2

In Experiment 1, we replicated previous observations of a positive evidence bias in discrimination confidence, such that confidence scaled with the total sum of evidence for both hypotheses. In contrast, in detection an effect of sum evidence was apparent for the decision, but not for the confidence kernels. Furthermore, confidence in detection ‘no’ responses was unaffected by fluctuations in motion energy.

Importantly, our analysis treated energy in the leftward and rightward directions as two independently represented quantities. Although models of motion perception commonly include such direction-selective sensory channels (Adelson & Bergen, 1985; Levinson & Sekuler, 1975; Van Santen & Sperling, 1984), it is unclear to what degree left and right motion energy channels are available to decision-making modules, as opposed to a mere subtraction between the two. In Exp. 2, the two sensory channels corresponded to two separate stimuli, making it much more likely that subjects represented them in an independent manner. Using these stimuli, we tested the generalizability of these findings to a different type of stimuli (flickering patches) and mode of data collection (a ~10-minute online experiment). Our preregistered objectives (documented here: https://osf.io/d3vkm/) were (1) to replicate a positive evidence bias in discrimination confidence, (2) to replicate the absence of a positive evidence bias in detection confidence, and (3) to replicate the absence of an effect of evidence on confidence in ‘no’ judgments.

### Methods

#### Participants

The research complied with all relevant ethical regulations, and was approved by the Research Ethics Committee of University College London (study ID number 1260/003). 147 participants were recruited via Prolific (prolific.co) and gave their informed consent prior to their participation. They were selected based on their acceptance rate (>95%) and for being native English speakers. Following our preregistration, we aimed to collect data until we had reached 100 included participants based on our prespecified inclusion criteria (see https://osf.io/d3vkm/). Our final data set includes observations from 102 included participants. The entire experiment took around 10 minutes to complete. Participants were paid £1.25 for their participation, equivalent to an hourly wage of £7.50.

#### Experimental paradigm

A static demo of Exp. 2 is available on the project’s GitHub. The experiment was programmed using the jsPsych and P5 JavaScript packages (De Leeuw, 2015; McCarthy, 2015), and was hosted on a JATOS server (Lange et al., 2015). It consisted of two tasks (Detection and Discrimination) presented in separate blocks. A total of 56 trials of each task was delivered in two blocks of 28 trials each. The order of experimental blocks was interleaved, starting with discrimination.

The first discrimination block started after an instruction section, which included instructions about the stimuli and confidence scale, four practice trials and four confidence practice trials. Further instructions were presented before the second block. Instruction sections were followed by multiple-choice comprehension questions, to monitor participants’ understanding of the main task and confidence reporting interface. To encourage concentration, in addition to trial-wise feedback we also provided participants with feedback about their overall performance and mean confidence at the end of the second and fourth blocks.

Importantly, unlike in the lab-based experiment, there was no calibration of difficulty for the two tasks. The rationale for this is that in Exp. 1, perceptual thresholds for motion discrimination were highly consistent across participants, and staircasing took a long time to converge. Furthermore, in Exp. 1, we aimed to control for task difficulty, but this introduced differences between the stimulus intensities used for detection and discrimination. To complement our findings, here we aimed to match stimulus intensity between the two tasks and accepted that task performance might vary between detection and discrimination as a result.

#### Trial structure

**Fig. 7:**
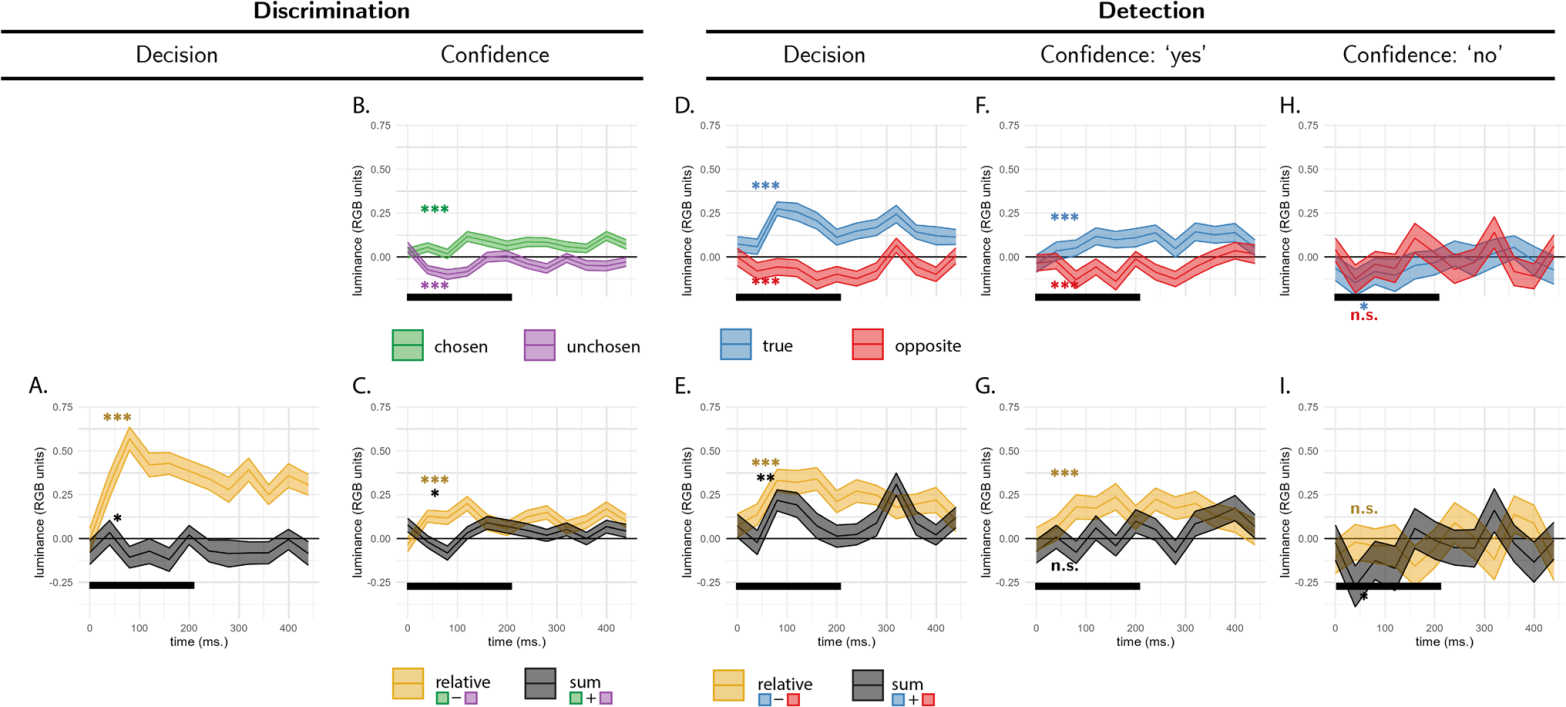
Reverse correlation, Exp. 2. Same conventions as in Fig. 5. (Colour figure online)

In discrimination blocks, trial structure closely followed Exp. 2 from Zylberberg et al. (2012), with a few adaptations. Following a fixation cross (500 ms), two sets of four adjacent vertical gray bars were presented as a rapid serial visual presentation (RSVP; 12 frames, presented at 25 Hz), displayed to the left and right of the fixation cross (see Fig. 7). On each frame, the luminance of each bar was randomly sampled from a Gaussian distribution with a standard deviation of 10/255 units in the standard RGB 0-255 coordinate system. For one set of bars, this Gaussian distribution was centered at the same luminance value as the background (128/255). For the other set, it was centered at 133/255, making it brighter on average. Participants then reported which of the two sets was brighter on average using the ‘D’ and ‘F’ keys on the keyboard. After their response, they rated their confidence on a continuous scale, by controlling the size of a coloured circle with their mouse. High confidence was mapped to a big, blue circle, and low confidence to a small, red circle. To discourage hasty confidence ratings, the confidence rating scale stayed on the screen for at least 2,000 ms. Feedback about decision accuracy was delivered after the confidence rating phase.

Detection blocks were similar to discrimination blocks, with the exception that decisions were made about whether the average luminance of either of the two sets was brighter than the gray background, or not. In ‘different’ trials, the luminance of the four bars in one of the sets was sampled from a Gaussian distribution with mean 133/255, and the luminance of the other set from a Gaussian distribution with mean 128/255. In ‘same’ trials, the luminance of both sets was sampled from a distribution centered at 128/255. Participants were told that only one of the two patches could be bright, but never both. Decisions in Detection trials were reported using the ‘Y’ and ‘N’ keys. Confidence ratings and feedback were as in the discrimination task.

### Results

#### Decision accuracy

Overall proportion correct was 0.85 in the discrimination and 0.67 in the detection task. Performance in discrimination was significantly higher than in detection (*M*_*D*_ = 0.18), 95% CI [0.16, 0.20], *t*(101) = 18.01, *p* < .001. Unlike in Exp. 1, where we aimed to control for task difficulty, here we decided to match stimulus intensity between the two tasks, so a difference between detection and discrimination performance was expected (Wickens, 2002).

#### Overall properties of decision and confidence distributions

Similar to Exp. 1, participants were more likely to respond ‘yes’ than ‘no’ in the detection task (mean proportion of ‘yes’ responses: 0.54). We did not observe a consistent response bias in discrimination (mean proportion of ‘right’ responses: 0.50). The two detection responses showed the typical asymmetries, with ‘yes’ responses being faster (median difference of 77 ms) and accompanied by higher levels of confidence (mean difference of 0.10 on a 0–1 scale). Unlike in Exp. 1, here we found no evidence for a difference in metacognitive sensitivity between ‘yes’ and ‘no’ responses (mean difference of 0.02 in AUC units). No asymmetries were observed between the two discrimination responses. For a detailed statistical analysis, see Appendix.

#### Reverse correlation

Stimuli in Exp. 2 consisted of two flickering patches, each comprising four gray bars presented for 12 frames. Together, this summed to 96 random luminance values per trial, which we subjected to reverse correlation analysis, following the analysis procedure of Exp. 2 in Zylberberg et al. (2012).

#### Discrimination decisions

First, we asked whether random fluctuations in luminance influenced discrimination responses. Similar to the results obtained by Zylberberg et. al., discrimination decisions were sensitive to fluctuations in relative evidence (the difference in mean luminance between the left and right stimulus) during the first 300 ms of the trial (*E*_*relative*_; *t*(100) = 14.29, *p* < .001; see Fig. 7A, orange curve). Furthermore, participants’ decisions were surprisingly more sensitive to evidence in the nontarget stimulus within the same time window, resulting in a negative effect of sum evidence (*E*_*sum*_; *t*(100) =  − 2.29, *p* = .024; see Fig. 7A, black curve). Importantly, this negative effect of sum evidence on decision accuracy was not replicated in Exps. 3 and 4, and we do not interpret it further.

#### Discrimination confidence

Similar to Exp. 1, we observed a significant effect of positive (*E*_*conf* − *chosen*_; *t*(100) = 6.76, *p* < .001), and negative (*E*_*conf* − *unchosen*_; *t*(100) =  − 4.28, *p* < .001), evidence on decision confidence within the first 300 ms of the stimulus (see Fig. 7B). When plotting the sum-evidence kernel, we observed an initial negative dip followed by a sustained positive effect of sum evidence on decision confidence, consistent with a positive evidence bias (*E*_*conf* − *sum*_; *t*(100) = 2.56, *p* = .012; see Fig. 7C, black curve).

#### Detection

Participants’ detection decisions were sensitive to fluctuations in the luminance of the target stimulus, such that ‘yes’ responses were associated with a brighter target stimulus (*E*_*detection* − *s*_; *t*(101) = 9.39, *p* < .001; see Fig. 7D, blue curve). Similar to Exp. 1, and in contrast to the behaviour of Bayes-rational simulated agents, the luminance of the nontarget stimulus had a negative effect on the probability of responding ‘yes’ (*E*_*detection* − *n*_; *t*(101) =  − 4.64, *p* < .001; see Fig. 7D, red curve).

Confidence in detection ‘yes’ responses was similarly sensitive to fluctuations in the luminance of the target stimulus (*E*_*conf* − *yes* − *s*_; *t*(99) = 4.27, *p* < .001; see Fig. 7F, blue curve). Again, brighter nontarget stimuli made participants less, rather than more, confident in the presence of a signal (*E*_*conf* − *yes* − *n*_; *t*(99) =  − 4.98, *p* < .001; see Fig. 7F, red curve). As in Exp. 1, here, too, sum evidence (overall luminance) had no significant effect on confidence in detection ‘yes’ responses (*E*_*conf* − *yes* − *sum*_; *t*(99) =  − 0.28, *p* = .784; see Fig. 7G, black curve). However, this surprising result was not replicated in Experiments 3 and 4.

Finally, unlike in Exp. 1, confidence in detection ‘no’ responses was sensitive to random fluctuations in the luminance of the target, such that participants were more confident in the absence of a signal when the target stimulus was darker (*E*_*conf* − *no* − *s*_ *t*(96) =  − 2.28, *p* = .025; see Fig. 7H). The overall luminance of the display also had a negative effect on confidence in detection ‘no’ responses (*E*_*conf* − *no* − *sum*_; *t*(96) =  − 2.04, *p* = .044; see Fig. 7I). The luminance of the nontarget stimulus (*E*_*conf* − *no* − *n*_; *t*(96) =  − 0.71, *p* = .482), and the difference in luminance between the two stimuli (*E*_*conf* − *no* − *relative*_; *t*(96) =  − 1.04, *p* = .301), had no significant effects on confidence in detection ‘no’ responses.

## Experiment 3

In Exp. 3, we aimed to replicate our findings using a direct experimental manipulation in addition to employing reverse-correlation analysis. Our preregistered objectives (see our preregistration document: https://osf.io/hm3fn/) were (1) to replicate a positive evidence bias in discrimination confidence, (2) to replicate a positive evidence bias in detection decisions, and (3) to replicate the absence of a positive evidence bias in detection confidence.

### Methods

#### Participants

The research complied with all relevant ethical regulations, and was approved by the Research Ethics Committee of University College London (study ID number 1260/003). A total of 173 participants were recruited via Prolific (prolific.co) and gave their informed consent prior to their participation. They were selected based on their acceptance rate (>95%) and for being native English speakers. Following our preregistration, we aimed to collect data until we had reached 100 included participants, based on our prespecified inclusion criteria (see https://osf.io/hm3fn/). Our final data set includes observations from 100 included participants. The entire experiment took around 20 minutes to complete. Participants were paid £2.50 for their participation, equivalent to an hourly wage of £7.50.

#### Experimental paradigm

A static demo of Exp. 3 is available on the project’s GitHub. Experiment 3 was identical to Experiment 2 with two changes. First, on half of the trials (high-luminance trials) the luminance of both sets of bars was increased by 2/255 for the entire duration of the display, thereby increasing sum evidence without affecting relative evidence. Second, in order to increase our statistical power for detecting response-specific effects in detection, participants performed four detection blocks and two discrimination blocks. Each block comprised 56 trials. The order of blocks was [detection, discrimination, detection, discrimination, detection, detection] for all participants.

### Results

#### Decision accuracy

Overall proportion correct was 0.87 in the discrimination and 0.67 in the detection task. Performance in discrimination was significantly higher than in detection (*M*_*D*_ = 0.21), 95% CI [0.19, 0.22], *t*(99) = 30.35, *p* < .001, as expected.

#### Overall properties of decision and confidence distributions

Similar to Exps. 1 and 2, participants were more likely to respond ‘yes’ than ‘no’ in the detection task (mean proportion of ‘yes’ responses: 0.53). We did not observe a consistent response bias in the discrimination task (mean proportion of ‘right’ responses: 0.50). The two detection responses showed the typical asymmetries, with ‘yes’ responses being faster (median difference of 69 ms) and accompanied by higher levels of confidence (mean difference of 0.09 on a 0–1 scale). As in Exp. 1, metacognitive sensitivity was higher for ‘yes’ than for ‘no’ responses (mean difference of 0.03 in AUC units). No asymmetries were observed between the two discrimination responses. For a detailed statistical analysis, see Appendix.

#### Reverse correlation

We first focused on reverse correlation analyses, pooling data from both high-luminance and standard trials (after mean-centering luminance in each), in order to replicate the findings of Exps. 1 and 2. We note the results are qualitatively similar when including standard trials only, with the exception of confidence in detection ‘no’ responses (see Appendix).

#### Discrimination decisions

**Fig. 8:**
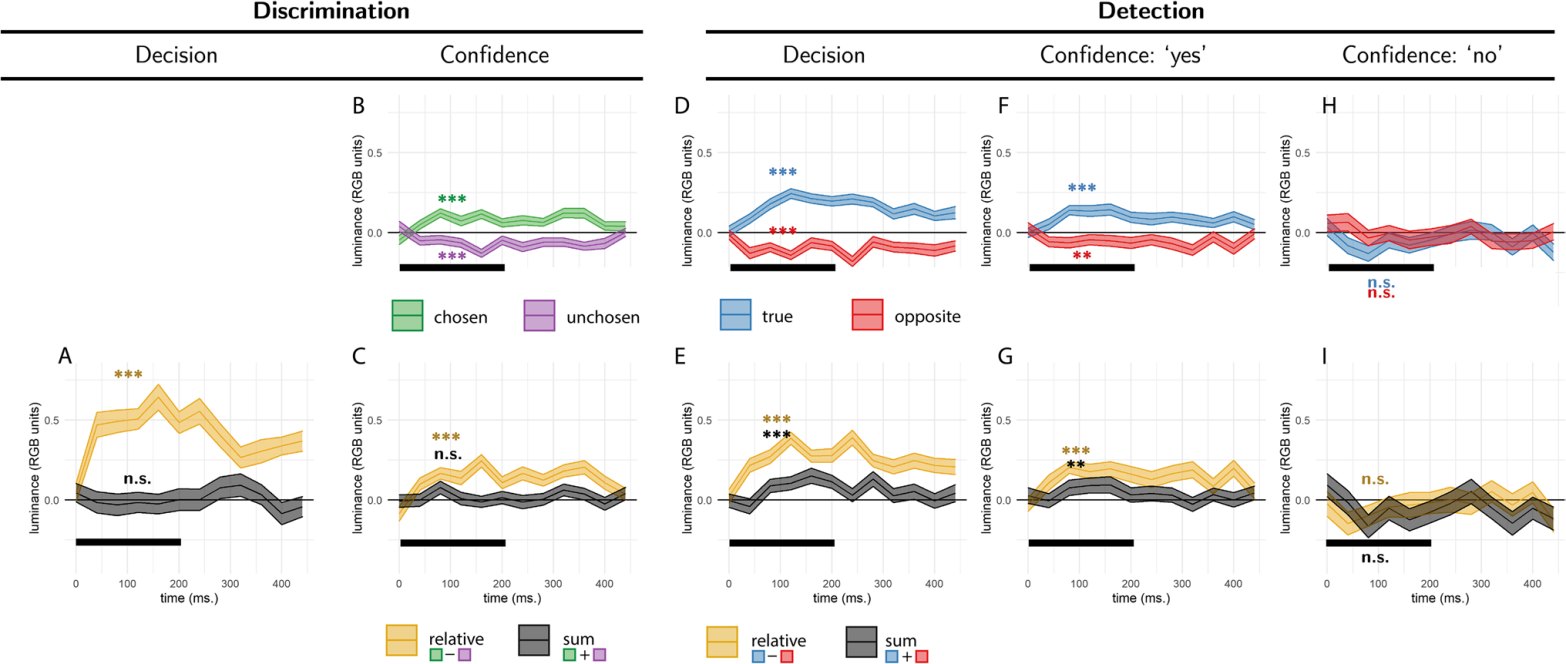
Reverse correlation, Exp. 3. Same conventions as in Fig. 5. (Colour figure online)

Discrimination decisions were sensitive to relative evidence during the first 300 ms of the trial (*E*_*relative*_; *t*(99) = 19.17, *p* < .001; see Fig. 8A) with no effect of sum evidence (*E*_*sum*_;*t*(99) = 0.23, *p* = .817).

#### Discrimination confidence

Decision confidence was sensitive to positive (*E*_*conf* − *s*_; *t*(99) = 6.27, *p* < .001) and negative (*E*_*conf* − *n*_; *t*(99) =  − 5.29, *p* < .001) evidence within the first 300 ms of the stimulus (see Fig. 8B). Reverse correlation revealed no effect of random fluctuations in sum evidence on decision confidence (*E*_*conf* − *sum*_; *t*(99) = 0.75, *p* = .455), but an effect of sum evidence was found when directly contrasting high- and low-luminance trials, as we show in the “Evidence-weighting” section below.

#### Detection

Participants’ detection decisions were sensitive to fluctuations in the luminance of the target stimulus, such that ‘yes’ responses were associated with brighter target stimuli (*E*_*detection* − *s*_; *t*(99) = 13.01, *p* < .001; see Fig. 8D, blue curve). Replicating the surprising results of Exps. 1 and 2, the luminance of the nontarget stimulus had a negative effect on the probability of responding ‘yes’ in the detection task (*E*_*detection* − *n*_; *t*(99) =  − 8.91, *p* < .001; see Fig. 8D, red curve). Together, detection decisions were sensitive to relative evidence (*E*_*detection* − *relative*_, or the difference in luminance between the target and nontarget stimuli; *t*(99) = 15.95, *p* < .001), and to sum evidence (*E*_*detection* − *relative*_, or the overall luminance of the display; *t*(99) = 4.29, *p* < .001; see Fig. 8E, orange and black curves, respectively).

Confidence in detection ‘yes’ responses was similarly positively correlated with the luminance of the target stimulus (*E*_*conf* − *yes* − *s*_; *t*(99) = 8.37, *p* < .001), and negatively correlated with the luminance of the nontarget stimulus (*E*_*conf* − *yes* − *s*_; *t*(99) =  − 3.58, *p* = .001; see Fig. 8F). This is again in contrast to what is expected from a Bayes-rational agent: the probability of being correct is positively correlated with evidence intensity in both signal and nonsignal channels. Recall that a surprising finding in Exps. 1 and 2 was that sum evidence (motion energy or luminance) had no effect on participants’ confidence in their judgments of stimulus presence. In contrast, in Exp. 3, sum luminance had a significant positive effect on decision confidence when reporting target presence (*E*_*conf* − *yes* − *sum*_; *t*(99) = 2.83, *p* = .006; see Fig. 8G, black curve).

Finally, and in line with what we observed in Exp. 2, confidence in detection ‘no’ responses was sensitive to random fluctuations in the luminance of the target, such that participants were more confident in the absence of a signal when the target stimulus was darker (*E*_*conf* − *no* − *s*_; *t*(98) =  − 2.72, *p* = .008; see Fig. 8H). Relative evidence also had a marginally significant negative effect on confidence in decisions about absence (*E*_*conf* − *no* − *relative*_; *t*(98) =  − 1.98, *p* = .050). The luminance of the nontarget stimulus and the overall luminance had no significant effects on confidence in detection ‘no’ responses (*p*s > 0.3).

#### Evidence-weighting

In Experiments 1 and 2, confidence in signal presence was invariant to sum evidence (overall motion energy in Exp.1, sum luminance in Exp. 2). This was surprising for two reasons. First, in both cases sum evidence did have a significant effect on detection decisions. Second, incorporating information about sum evidence into confidence in the presence of a stimulus is rational: A target stimulus is more likely to be present (in either location) when both target *and* nontarget stimuli are brighter compared with when both are dark. As we document above, however, the counterintuitive findings of Exps. 1 and 2 only partly replicated in Exp. 3: subjects still negatively weighted the luminance of the nontarget stimulus (despite this being irrational), but this negative effect was weaker than the positive effect of the luminance of the target stimulus, resulting in an overall positive effect of sum evidence on detection confidence.

To shed further light on this issue, in Exp. 3, half of the trials were manipulated to include slightly brighter stimuli, thereby increasing statistical power for tests of the effects of sum luminance on discrimination and detection decisions and confidence.

First, we established that participants were more likely to respond ‘yes’ on higher compared with lower luminance trials (*M* = 0.09), 95% CI [0.07, 0.11], *t*(99) = 8.73, *p* < .001, consistent with overall luminance providing a valid cue for signal presence.

**Fig. 9:**
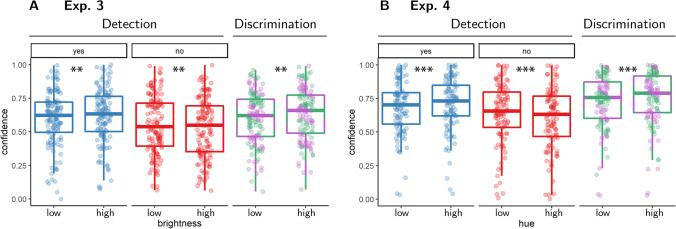
Difference in confidence between standard and higher evidence (luminance and hue) trials for the three response categories (detection ‘yes’ and ‘no’ responses, and discrimination responses) in Exps. 3 and 4. Box edges and central lines represent the 25, 50, and 75 quantiles. Whiskers cover data points within four interquartile ranges around the median. Stars represent significance in a two-sided *t* test: **p < .01, ***p < .001. (Colour figure online)

We next turned to the effects of our luminance manipulation on confidence. For discrimination judgments, participants were also more confident in higher compared with lower luminance trials (*M* = 0.02), 95% CI [0.01, 0.03], *t*(99) = 3.20, *p* = .002 (see Fig. 9A), replicating a positive evidence bias in discrimination confidence. For detection judgments, in line with the reverse correlation analysis of Exp. 3 (and in contrast to the findings of Experiments 1 and 2), participants were more confident in their ‘yes’ responses when overall luminance was higher (*M* = 0.02), 95% CI [0.01, 0.03], *t*(99) = 3.00, *p* = .003. Our preregistered Bayesian analysis provided strong evidence for the alternative hypothesis that detection confidence is affected by this manipulation (BF_10_ = 10.57). Furthermore, this increase in confidence in presence as a function of the brightness manipulation was not significantly different from that observed for discrimination confidence (*M* =  − 0.01), 95% CI [−0.03, 0.01], *t*(99) =  − 0.57, *p* = .573. Finally, and in line with Exp. 2, overall luminance had a significant negative effect on confidence in ‘no’ responses (*M* =  − 0.02), 95% CI [−0.03, −0.01], *t*(99) =  − 3.09, *p* = .003, indicating that participants were more confident in the absence of a target when overall luminance was lower.

## Experiment 4

A limitation of Exps. 2 and 3 is that apparent asymmetries in the weighting of positive and negative evidence may result from a nonlinear mapping between luminance in RGB space and screen brightness.^2^ For example, a dark bar that is −2 RGB units from the mean does not necessarily cancel out a bright bar that is +2 RGB units from the mean (unless working with a gamma-corrected monitor), making positive evidence objectively more salient than negative evidence.

To address this concern, we include an additional experiment where evidence is sampled from a perceptually uniform space. Specifically, Exp. 4 was similar to Exp. 3 with the exception that flickering stimuli varied in their hue rather than luminance, and where hue values were sampled from a Gaussian distribution in the CIE L*a*b* colour space. Moreover, the roles of ‘target’ and ‘nontarget’ hues were counterbalanced between participants, such that any built-in asymmetries in the perception of positive and negative evidence should cancel out at the group level.

### Methods

#### Participants

The research complied with all relevant ethical regulations, and was approved by the Research Ethics Committee of University College London (study ID number 1260/003). A total of 117 participants were recruited via Prolific (prolific.co), and gave their informed consent prior to their participation. They were selected based on their acceptance rate (>95%) and for being native English speakers. Following our preregistration, we aimed to collect data until we had reached 100 included participants, based on our prespecified inclusion criteria (see https://osf.io/9zbpc). Our final data set includes observations from 100 included participants. The entire experiment took around 20 minutes to complete. Participants were paid £2.50 for their participation, equivalent to an hourly wage of £7.50.

#### Experimental paradigm

A static demo of Exp. 4 is available on the project’s GitHub. Experiment 4 was identical to Experiment 3, with two changes. First, flickering bars varied in hue, randomly sampled from a Gaussian distribution in the CIE L*a*b* colour space, centred at *L* = 54, *a* = 21.5 and *b* = 11.5, with a radius of 49 (Schurgin et al., 2020). For half of the participants, nontarget hues were sampled around an orientation of 1.85 radians with a standard deviation of 0.35, and target hues were sampled around an orientation of 2.1 with a standard deviation of 0.35. For the first group, target patches were little more orange than nontarget patches, and for the second group target patches were little more green than nontarget patches. To make sure nontarget patches were perceived as the absence of signal relative to the background, the RSVP display was overlaid on top of a rectangle with the mean colour of a nontarget patch. Second, in order to avoid interference with the colour-judgment task, the confidence circle was presented in gray. Third, subjects were allowed to repeat the multiple-choice questions up to three times. Finally, in addition to trial-wise feedback, block-wise feedback about overall performance and mean confidence in correct and incorrect responses was displayed at the end of each block.

### Results

#### Decision accuracy

Overall proportion correct was 0.92 in the discrimination and 0.74 in the detection task. Performance in discrimination was significantly higher than in detection (*ΔM* = 0.18), 95% CI [0.16, 0.20], *t*(199.32) = 16.52, *p* < .001, as expected.

#### Overall properties of decision and confidence distributions

Similar to Exps. 1–3, participants were more likely to respond ‘yes’ than ‘no’ in the detection task (mean proportion of ‘yes’ responses: 0.52). A slight response bias in discrimination was not significant (mean proportion of ‘right’ responses: 0.51). The two detection responses showed the typical asymmetries, with ‘yes’ responses being faster (median difference of 48 ms) and accompanied by higher levels of confidence (mean difference of 0.07 on a 0–1 scale). A mean difference of 0.03 in metacognitive sensitivity (AUC units) was not significant. For a detailed statistical analysis, see Appendix.

#### Reverse correlation

##### Discrimination decisions

**Fig. 10:**
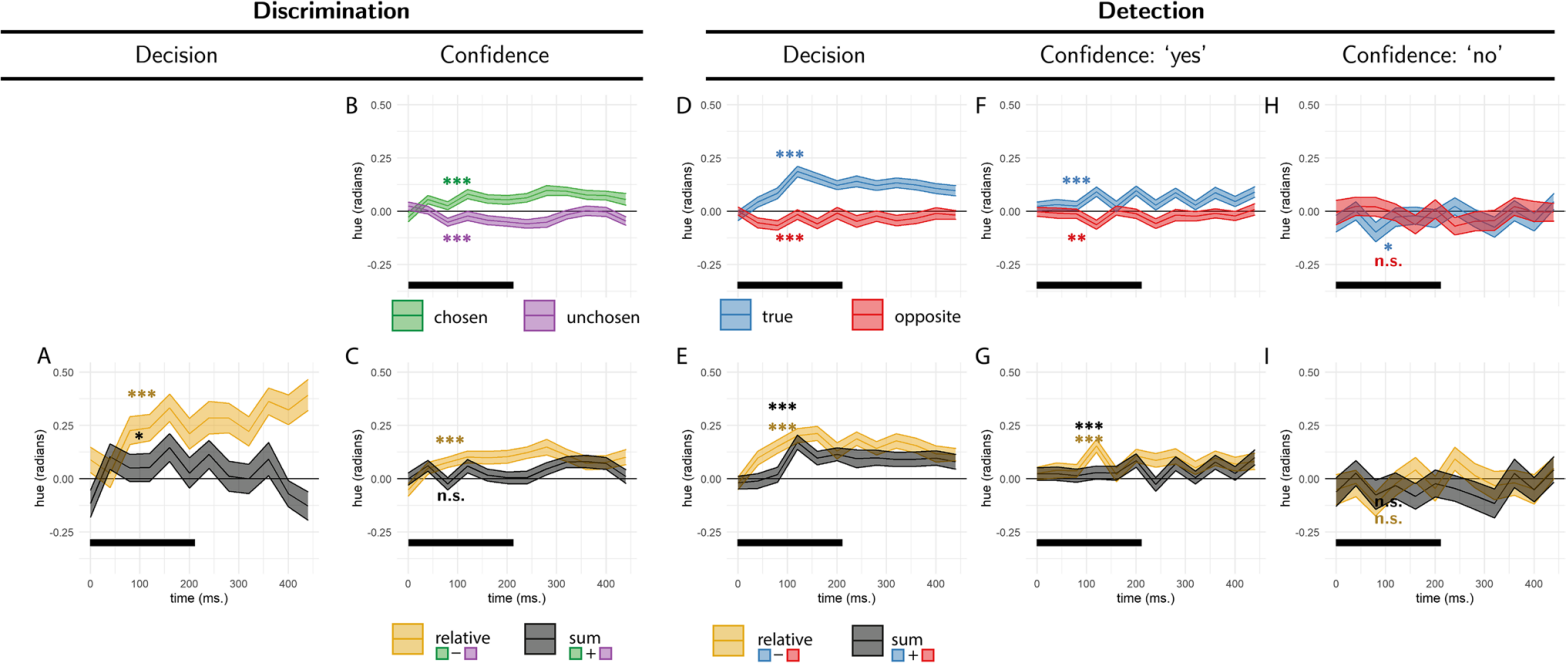
Reverse correlation, Exp. 4. Same conventions as in Fig. 5. (Colour figure online)

Discrimination decisions were sensitive to relative evidence during the first 300 ms of the trial (*E*_*relative*_; *t*(102) = 10.23, *p* < .001; see Fig. 10A). Sum evidence had a positive effect on discrimination decisions, such that subjects were more likely to correctly select the target stimulus when the overall hue of both stimuli together was closer to the target hue (*E*_*sum*_; *t*(102) = 2.31, *p* = .023).

##### Discrimination confidence

Decision confidence was sensitive to positive (*E*_*conf* − *chosen*_; *t*(108) = 6.00, *p* < .001) and negative (*E*_*conf* − *unchosen*_ ;*t*(108) =  − 3.94, *p* < .001) evidence within the first 300 ms (see Fig. 10B). The effect of sum evidence on decision confidence was only marginally significant (*E*_*conf* − *sum*_; *t*(108) = 1.92, *p* = .058). An effect of sum evidence was found when directly contrasting high- and low-evidence trials, as we show in the “Evidence-weighting” section below.

##### Detection

Participants’ detection decisions were sensitive to fluctuations in the hue of the target stimulus (*E*_*detection* − *s*_; *t*(101) = 10.65, *p* < .001; see Fig. 10D, blue curve). Fluctuations in the hue of the nontarget stimulus had the opposite effect on detection decisions, replicating again our main finding from Exps. 1–3 (*E*_*detection* − *n*_; *t*(101) =  − 4.22, *p* < .001; see Fig. 10D, red curve).

Confidence in detection ‘yes’ responses was positively sensitive to fluctuations in the hue of the target (*E*_*conf* − *yes* − *s*_; *t*(99) = 6.08, *p* < .001), and negatively sensitive to fluctuations in the hue of the nontarget stimulus (*E*_*conf* − *yes* − *n*_; *t*(99) =  − 2.63, *p* = .010; see Fig. 13F). Similar to Exp. 3, here, too, sum evidence (deviation from the background hue toward the target hue) had a significant positive effect on decision confidence when reporting target presence (*E*_*conf* − *yes* − *sum*_; *t*(99) = 2.64, *p* = .010; see Fig. 10G).

Finally, confidence in detection ‘no’ responses was sensitive to random fluctuations in the hue of the target, such that participants were more confident in the absence of a signal when the target stimulus was closer in hue to the background (*E*_*conf* − *no* − *s*_; *t*(97) =  − 2.23, *p* = .028; see Fig. 10H). Sum evidence (the overall hue of the display) had a marginal negative effect on confidence in absence (*t*(97) =  − 1.95, *p* = .054). Relative evidence and negative evidence had no significant effects on confidence in detection ‘no’ responses (*p*s > 0.3).

##### Evidence-weighting

As in Exp. 3, on half of the trials (‘high-evidence’ trials), the hue of both patches was slightly shifted in the direction of the target stimulus (that is, made greener if the target stimulus was greener than the nontarget stimulus, or more orange otherwise). This allowed us to directly measure how sum evidence affects both detection decisions, and detection and discrimination confidence ratings. Overall, we obtained a similar pattern to Exp. 3: on high-evidence trials, participants were more likely to respond ‘yes’ in the detection task (*M* = 0.17, 95% CI [0.15, 0.19], *t*(101) = 17.39, *p* < .001), and became more confident in their discrimination judgments (*M* = 0.02, 95% CI [0.01, 0.03], *t*(109) = 3.75, *p* < .001), more confident in their detection ‘yes’ responses (*M* = 0.03, 95% CI [0.02, 0.04], *t*(101) = 4.76, *p* < .001), and less confident in detection ‘no’ responses (*M* =  − 0.04, 95% CI [−0.05, −0.03], *t*(101) =  − 6.34, *p* < .001; see Fig. 9B). Our preregistered Bayesian analysis provided strong evidence for the alternative hypothesis that detection confidence is affected by sum evidence (BF_10_ = 2547.94). One difference in comparison to Exp. 3 was that in Exp. 4 the increase in ‘yes’ response confidence as a function of the hue manipulation was significantly stronger than that observed for discrimination confidence (*M* =  − 0.02, 95% CI [−0.04, −0.01], *t*(101) =  − 2.61, *p* = .011).

### Discussion

In four experiments, we compared the drivers of decisions and confidence ratings in perceptual discrimination and detection, in conditions either matched for difficulty (Exp. 1) or signal strength (Exps. 2–4). In order to measure the contribution of perceptual evidence to confidence in detection and discrimination judgments, we followed Zylberberg et al. (2012) in applying reverse correlation analysis to noisy stimuli in perceptual decision-making tasks. We fully replicated the main results of Zylberberg and colleagues: decisions and confidence were affected by perceptual evidence in the first 300 ms of the trial, peaking at around 200 ms. We also successfully replicated a positive evidence bias (PEB) for discrimination confidence: confidence in the discrimination task was more affected by supporting than by conflicting evidence—a pattern which may be indicative of a detection-like decision rule operating for discrimination confidence. This effect was qualitatively accounted for by two Bayes-rational decision-making models: a firing rate model, in which perceptual noise was stimulus dependent (inspired by Miyoshi & Lau, 2020), and a goal-directed attention model (inspired by Sepulveda et al., 2020).

These same two models also made corresponding predictions for the detection task: When attempting to detect signal presence in either channel, decisions and confidence ratings should positively weigh evidence for both alternatives (e.g., motion energy to the right and to the left). Paradoxically, however, in the detection task subjects adopted a discrimination-like disposition, negatively weighing evidence in the nonsignal channel. In other words, subjects were *less* likely to say a target was present (in either channel) when the weaker channel had *more* evidence. In Exps. 1 and 2, this negative weighting of evidence in the nonsignal channel was strong enough to bring the effect of sum evidence on detection confidence down to zero (as the surprising negative effect of nonsignal evidence canceled out the expected positive influence of signal evidence on detection probability). This negative weighting of evidence in the nonsignal channel remained significant in Experiments 3 and 4, although was now somewhat weaker than the positive weighting of the evidence in the signal channel, leading to an overall sum evidence effect on detection probability. Overall, then, subjects incorporated detection-relevant evidence into their confidence in discrimination judgments, and discrimination-relevant evidence into their detection judgments and confidence ratings.

What drives these discrimination-like evidence weighting profiles in detection? In Experiments 3 and 4, one explanation is that our evidence-boost manipulation may have rendered it rational for subjects to focus on the difference in evidence between the two sensory channels. If on a random subset of trials both stimuli are made brighter, focusing on overall brightness is not as informative as focusing on the contrast between the brightness of the two stimuli, which remains unaffected by the evidence-boost manipulation. This account fails to explain, however, the emergence of a negative effect of evidence in the nonsignal channel in Experiments 1 and 2, where the evidence-boost manipulation was not applied and where a rational agent should have positively weighted evidence from both channels.

Alternatively, changes to the global perception of overall stimulus intensity may have an internal source. For example, slow brain oscillations in the alpha band affect both detection criterion and discrimination confidence but have minimal effects on discrimination sensitivity: a nonselective effect on perception which has been attributed to a global change in the baseline firing rate of sensory neurons (Samaha et al., 2020). Similar to our evidence-boost manipulation, an overall increase in baseline firing rate increases sum evidence without affecting relative evidence. If agents do not have metacognitive access to the current excitability of their perceptual system but do know that such global effects exist, focusing on relative evidence in detection may be a rational way of dealing with this ambiguity of baseline excitability. This account fails to explain, however, why subjects in Exps. 2–4 did not use the static background rather than the nontarget stimulus as a reference point, given that it is presumably also susceptible to perceptual influences from global changes in the baseline firing rate of sensory neurons.

Finally, it may be that evidence accumulation in detection and discrimination depends on shared processes and internal representations. Outside of a laboratory setting, detectability and discriminability mostly go hand in hand; the farther away from ‘nothing’ a representation is, the more distinct and differentiated from other representations it becomes. Given these meta-level expectations about the distribution of evidence in the world, the overall availability of evidence may be a valid cue for confidence in discrimination judgments (Maniscalco et al., 2016). Conversely, asymmetries in the availability of evidence for two competing hypotheses may serve as a valid cue for the presence of signal in one of the channels.

If discrimination confidence and detection decisions are drawing on shared evidence weighting mechanisms, one might expect that person-specific tendencies to rely more on one or other evidence channel will be correlated across both tasks. For example, subjects whose discrimination confidence was strongly affected by sum evidence (or equivalently, showed a pronounced positive evidence bias), may also be sensitive to sum evidence in their detection decisions and confidence. Surprisingly, however, we find no evidence for such an effect (see Appendix). Across subjects, the effects of positive, negative, sum and relative evidence on discrimination confidence were not reliably correlated with their corresponding effects on detection decisions, nor with their effects on confidence in signal presence. This null result should be interpreted with caution: our experiments were not powered to identify correlations between participants, with Exp. 1 adopting a small-*N*, many-trials design, and Experiments 2–4 a high-*N*, few-trials design, with the attendant limitation of noisy single-subject estimates. Thus while our current results do not directly support a shared-resources account, they are not inconsistent with it.

### Conclusion

In four experiments, we replicated previous findings of a “positive evidence bias”: a detection-like evidence weighting in discrimination confidence. This pattern was accounted for by models that posit asymmetries either in the distributions of sensory noise or allocation of attention between target and nontarget channels. However, these same models could not account for a surprising finding of discrimination-like evidence weighting in detection decisions and confidence. We suggest that these seemingly irrational positive and negative evidence biases may reflect, at least in part, shared representational resources being harnessed for detection decisions and discrimination confidence.

## Figures and Tables

**Fig. A1:**
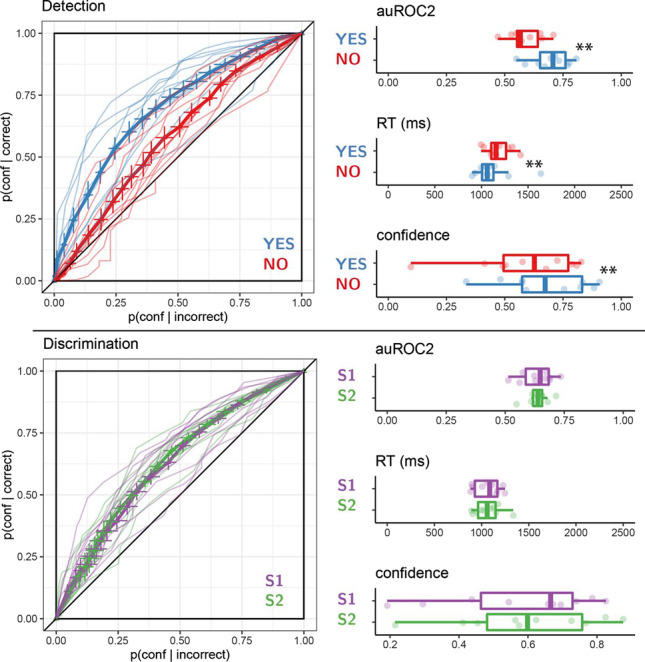
Behavioural asymmetries in metacognitive sensitivity, response time, and overall confidence in detection (upper panel) and discrimination (lower panel), in Exp. 1. Left: Response conditional Type 2 ROC curves for the two tasks and four responses in Exp. 1. The area under the Type 2 ROC curve is a measure of metacognitive sensitivity, and the difference in areas between the two responses a measure of metacognitive asymmetry. Single-subject curves are presented in low opacity. Right: Distributions of the area under the Type 2 ROC curve, median response time, and mean confidence for the four responses, across participants. Box edges and central lines represent the 25, 50, and 75 quantiles. Whiskers cover data points within four interquartile ranges around the median. Stars represent significance in a two-sided *t* test: **p < .01, ***p < .001. (Colour figure online)

**Fig. A2:**
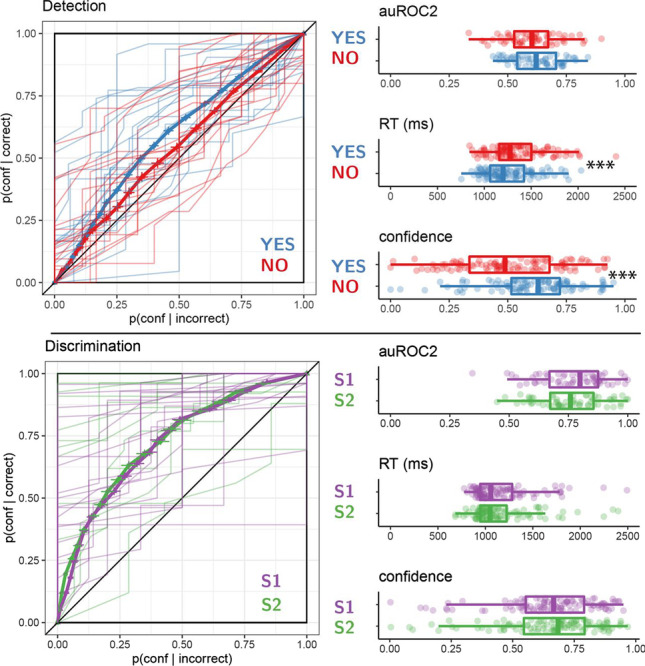
Behavioural asymmetries in metacognitive sensitivity, response time, and overall confidence, in Exp. 2. Same conventions as in Fig. A1. (Colour figure online)

**Fig. A3:**
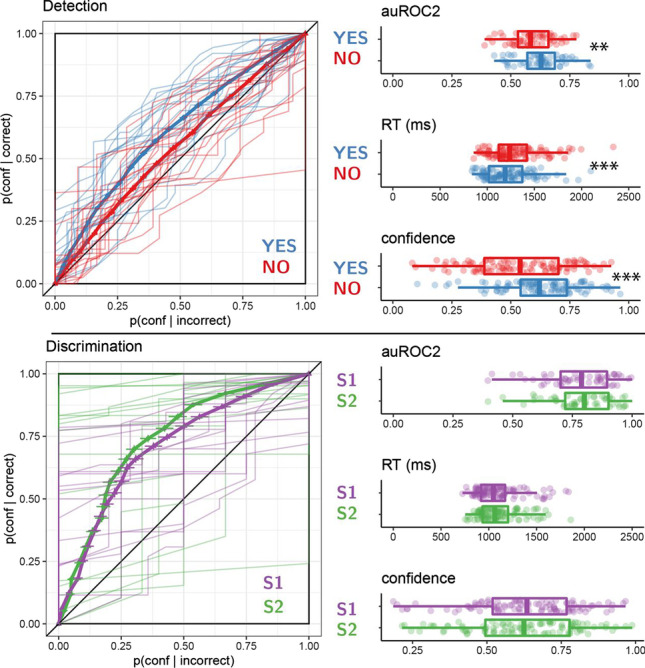
Behavioural asymmetries in metacognitive sensitivity, response time, and overall confidence, in Exp. 3. Same conventions as in Fig. A1. (Colour figure online)

**Fig. A4:**
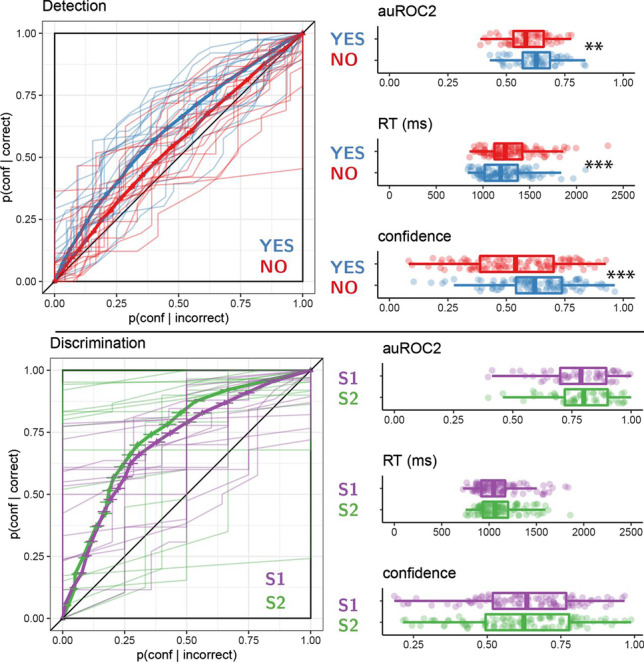
Behavioural asymmetries in metacognitive sensitivity, response time, and overall confidence, in Exp. 4. Same conventions as in Fig. A1. (Colour figure online)

**Fig. A5:**
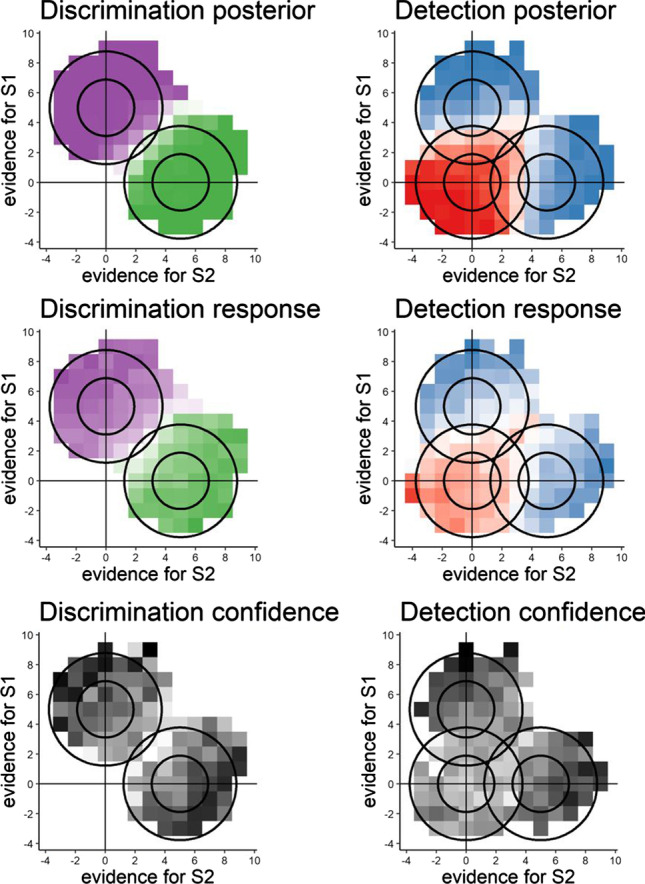
Top row: Posterior probability of stimulus category given perceptual evidence for discrimination (left) and detection (right). Middle row: Decision probability as a function of perceptual evidence. Bottom row: Mean confidence in correct responses as a function of perceptual evidence. (Colour figure online)

**Fig. A6:**
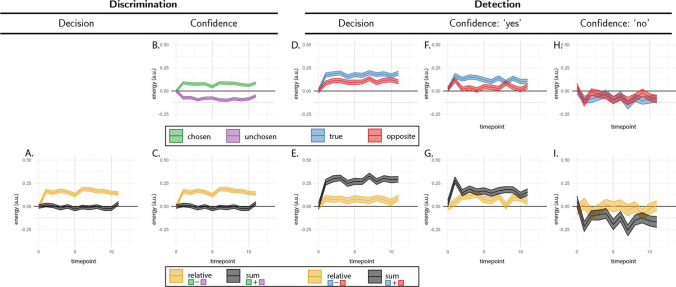
Reverse correlation kernels derived from simulation of the vanilla model. Same conventions as Fig. 5. (Colour figure online)

**Fig. A7:**
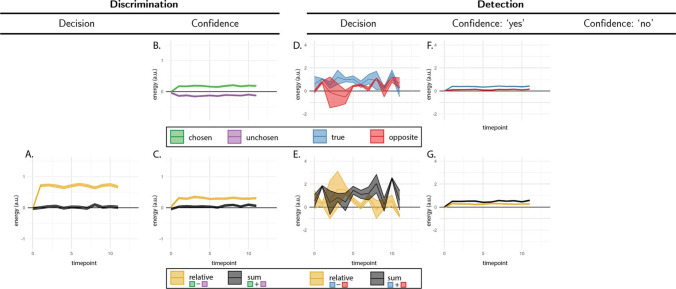
Reverse correlation kernels derived from simulation of the firing rate model. Same conventions as Fig. 5. (Colour figure online)

**Fig. A8:**
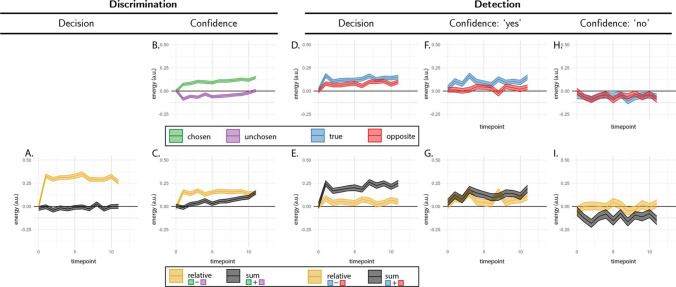
Reverse correlation kernels derived from simulation of the guided attention model. Same conventions as Fig. 5. (Colour figure online)

**Fig. A9:**
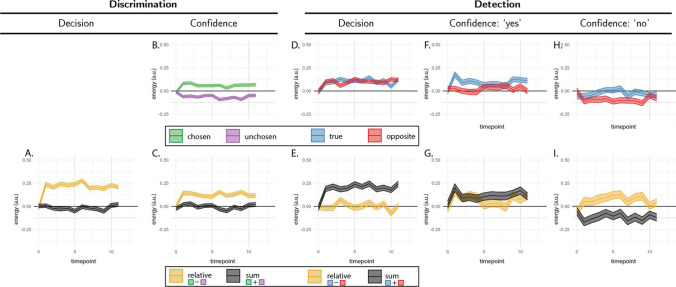
Reverse correlation kernels derived from simulation of the random attention model. Same conventions as Fig. 5. (Colour figure online)

**Fig. A10:**
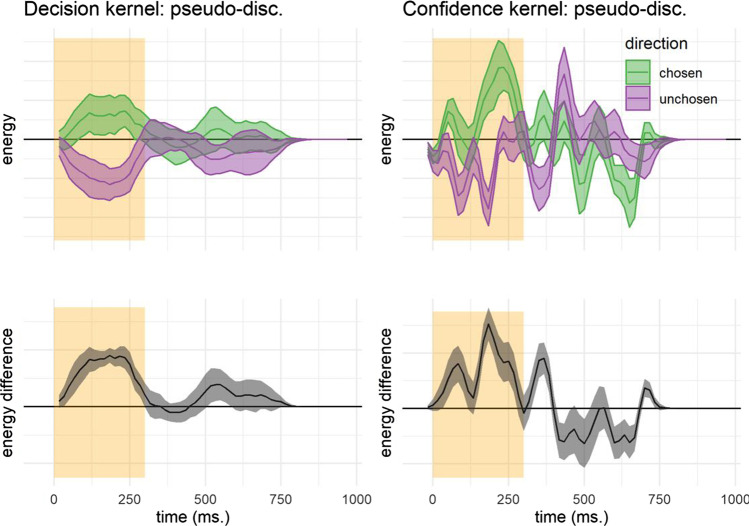
Decision and confidence pseudo-discrimination kernels, Experiment 1. Upper left: Motion energy in the ‘chosen’ (green) and ‘unchosen’ (purple) direction as a function of time. Bottom left: A subtraction between energy in the ‘chosen’ and ‘unchosen’ directions. Upper right: Confidence effects for motion energy in the ‘chosen’ (green) and ‘unchosen’ (purple) directions. Lower right: A subtraction between confidence effects in the ‘chosen’ and ‘unchosen’ directions. Shaded areas represent the mean ±1 standard error. The first 300 ms of the trial are marked in yellow. (Colour figure online)

**Fig. A11:**
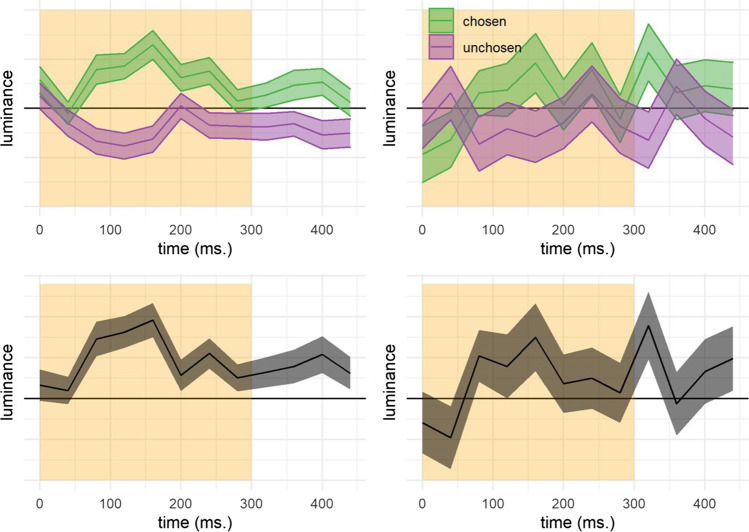
Decision and confidence pseudodiscrimination kernels, Experiment 2. Upper left: Luminance in the ‘chosen’ (green) and ‘unchosen’ (purple) stimulus as a function of time and spatial position. Bottom left: Decision kernel averaged across the four spatial positions. Upper right: Confidence effects for motion energy in the ‘chosen’ (green) and ‘unchosen’ (purple) stimuli. Bottom right: Confidence effects averaged across the four spatial positions. Shaded areas represent the mean ±1 standard error. The first 300 ms of the trial are marked in yellow. (Colour figure online)

## Appendix

### Additional analyses: Exp. 1

#### Response time, confidence, and metacognitive sensitivity differences

In detection, participants were generally slower to deliver ‘no’ responses compared with ‘yes’ responses (median difference: 85.37 ms, *t*(9) =  − 3.46, *p* = .007, for a *t* test on the log-transformed response times). No significant difference in response times was observed for the discrimination task (median difference: 6.16 ms, *t*(9) =  − 0.43, *p* = .676, see Fig. A1).

Confidence in detection was generally higher than in discrimination (*M*_*D*_ = 0.06, 95% CI [0.01, 0.12], *t*(9) = 2.49, *p* = .035). Within detection, confidence in ‘yes’ responses was generally higher than confidence in ‘no’ responses (*M* = 0.08; 95% CI [0.03, 0.13], *t*(9) = 3.49, *p* = .007). No difference in average confidence levels was found between the two discrimination responses (*M* = 0.02, 95% CI [−0.03, 0.06], *t*(9) = 0.91, *p* = .384).

Following Meuwese et al. (2014), we extracted response-conditional Type 2 ROC (rc-ROC) curves for the two tasks. For different values of *x* ∈ [0, 1], we plotted *p*(*confidence* ≥ *x*) for correct against incorrect responses, separately for the two response options. Unlike traditional Type 1 ROC curves that provide a summary of subjects’ ability to distinguish between two external world states, Type 2 ROC curves represent their ability to track the accuracy of one’s own responses. The area under the response-conditional ROC curve (auROC2) is a measure of metacognitive sensitivity, with higher values corresponding to more accurate metacognitive monitoring.

Mean response-conditional ROC curves for the two responses in the discrimination task closely matched (*M* = 0.00, 95% CI [−0.05, 0.05], *t*(9) = 0.13, *p* = .900), indicating that on average, participants had similar metacognitive insight into the accuracy of the two discrimination responses. In contrast, auROC2 estimates for ‘yes’ responses were significantly higher than for ‘no’ responses, indicating a metacognitive asymmetry between the two detection responses (group difference in auROC2: *M* = 0.11, 95% CI [0.03, 0.18], *t*(9) = 3.28, *p* = .010).

#### zROC curves

A difference in response-conditional auROC2 estimates can emerge from higher-order differences in metacognitive monitoring for the two responses and/or from lower-level differences in the perceptual representations of signal and noise (such as in first-order signal detection models where the signal variance is higher; Maniscalco & Lau, 2014). Importantly, a difference can also emerge in first-order signal-detection models that assume equal variance, in the presence of a response bias or insufficient variance in confidence ratings (Mazor et al., 2021). To test whether the metacognitive asymmetry between ‘yes’ and ‘no’ responses could be accounted for by an equal-variance SDT model, we simulated data that were identical to our empirical data except for confidence ratings in correct responses, which were chosen to perfectly agree with the assumptions of an equal-variance SDT model given participants’ decision criterion, sensitivity, and their confidence in incorrect responses. We then compared subject-wise differences between the response-conditional auROCs with the differences in this simulated data set (Mazor et al., 2021). The difference in differences was significant, indicating that the observed metacognitive asymmetry could not be accounted for by a first-order equal-variance SDT model (*M* = 0.08, 95% CI [0.02, 0.14], *t*(9) = 2.96, *p* = .016).

An asymmetry in metacognitive sensitivity for ‘yes’ and ‘no’ responses is also predicted by unequal-variance signal detection theory (*uvSDT*). Specifically, if the signal distribution is wider than the noise distribution, the overlap between the distributions will be more pronounced for misses and correct rejections than for hits and false alarms, making metacognitive judgments for ‘no’ responses objectively more difficult. Unequal-variance SDT predicts that plotting the Type 1 ROC curve in *z*-space (taking the inverse cumulative distribution of the confidence rating histogram) will result in a straight line with a slope equal to $\frac{\sigma_{noise}}{\sigma_{signal}}$. Because the variance of the signal distribution is higher than that of the noise distribution, zROC slopes are typically shallow, with slopes below 1.

To obtain an unbiased measure of zROC slopes while controlling for underestimation of the slope due to regression to the mean (Wickens, 2002, p. 56), we used total least squares estimation (Li et al., 2018). In equal-variance SDT, the natural logarithm of the zROC slope is predicted to be 0, corresponding to a slope of 1.

Detection zROC slopes were generally shallower than 1 (as predicted by an unequal-variance SDT model; *M* =  − 0.16, 95% CI [−0.28, −0.04], *t*(9) =  − 2.96, *p* = .016), and not significantly different from 1 for discrimination zROC curves (as predicted by equal-variance SDT; *M* = 0.00, 95% CI [−0.10, 0.10], *t*(9) = 0.06, *p* = .951).

These results support a difference in the variance-structure of the representation of signal and noise, such that the representation of signal is more variable across trials. However, it is still possible that some of the metacognitive asymmetry in detection (the difference in auROC2 between ‘yes’ and ‘no’ responses) reflects additional processes that cannot be captured by a first-order signal-detection model. If this was the case, zROC curves for detection should not only be shallower but also less linear than for discrimination, reflecting poorer fit of the signal-detection model to detection. To test if this was the case, we compared the subject-wise *R*^2^ values for the detection and discrimination zROC regression lines. *R*^2^ values reflect the goodness of fit of a linear model to the data. These values were similar for the two tasks (*M*_*D*_ =  − 0.01, 95% CI [−0.03, 0.01], *t*(9) =  − 0.91, *p* = .385), suggesting that a first-order SDT model accounted equally well for the data from both tasks.

#### Confidence–RT correlations

Following our preregistered analysis plan, we extracted a Spearman correlation coefficient between confidence and response times separately for the two tasks and four responses. We find a negative correlation in all four cases (discrimination responses: −0.40 and −0.39, detection ‘yes’: −0.41, detection ‘no’: −0.33). As hypothesized, this negative correlation was significantly attenuated in detection ‘no’ responses compared with detection ‘yes’ responses (tested with a one-tailed *t* test; *t*(9) =  − 1.97, *p* = .040). The difference in correlation strength between detection ‘no’ responses and discrimination responses was only marginally significant (*t*(9) =  − 1.68, *p* = .063).

#### Global metacognitive estimates

At the end of each 100-trial block, participants estimated their block-wise accuracy. Mean estimated accuracy was 0.71 for discrimination and 0.69. These figures are close to true correct response rates: 0.74 in discrimination and 0.72 in detection.

A difference of 0.02 between mean accuracy estimates for discrimination and detection was not significant at the group level (*t*(9) = 1.22, *p* = .254).

### Additional analyses: Exp. 2

#### Response time, confidence, and metacognitive sensitivity differences

Participants were slower to deliver ‘no’ responses compared with ‘yes’ responses (median difference: 77.12 ms, *t*(101) =  − 6.84, *p* < .001 for a *t* test on the log-transformed response times; see Fig. A2). No significant difference in response times was observed for the discrimination task (median difference: 10.90 ms; *t*(101) =  − 1.40, *p* = .165).

Confidence in detection was generally lower than in discrimination, consistent with lower accuracy in this task (*M*_*D*_ =  − 0.09; 95% CI [−0.11, −0.07], *t*(101) =  − 8.41, *p* < .001). Within detection, confidence in ‘yes’ responses was generally higher than confidence in ‘no’ responses (*M* = 0.10, 95% CI [0.07, 0.12], *t*(101) = 8.15, *p* < .001). No difference in average confidence levels was observed between the two discrimination responses (*M* = 0.00, 95% CI [−0.02, 0.02], *t*(101) =  − 0.03, *p* = .974).

In contrast to the results of Exp. 1, auROC2 values for ‘yes’ and ‘no’ responses were not significantly different (group difference in area under the response-conditional curve, auROC2: *M* = 0.02, 95% CI [−0.02, 0.06], *t*(58) = 1.13, *p* = .264). auROC2s were also not significantly different when controlling for Type 1 response and confidence biases (*M* = 0.01, 95% CI [−0.03, 0.05], *t*(58) = 0.59, *p* = .560).

#### zROC curves

Unlike in Experiment 1, detection zROC slopes were not significantly different from 1 (*M* =  − 0.04, 95% CI [−0.09, 0.01], *t*(100) =  − 1.53, *p* = .130), whereas discrimination zROC slopes were significantly shallower than 1 (*M* =  − 0.15, 95% CI [−0.30, −0.01], *t*(93) =  − 2.14, *p* = .035). This unexpected result indicates equal variance for the signal and noise distributions, but higher variance for targets presented on the right than on the left. Furthermore, a first-order SDT model fitted the data significantly better for the detection task than for the discrimination (difference in *R*^2^ for the two tasks: *M* = 0.15, 95% CI [0.12, 0.18], *t*(93) = 8.85, *p* < .001).

#### Confidence–RT correlations

Following our preregistered analysis plan, we extracted a Spearman correlation coefficient between confidence and response times separately for the two tasks and four responses. We find a negative correlation in all four cases (discrimination responses: −0.41 and −0.45, detection ‘yes’: −0.32, detection ‘no’: −0.21). This negative correlation was significantly attenuated in detection ‘no’ responses compared with detection ‘yes’ responses (tested with a one-tailed *t* test: *t*(100) =  − 3.78, *p* < .001). The difference in correlation strength between detection ‘no’ responses and discrimination responses was also significant, *t*(100) =  − 7.79, *p* < .001).

### Additional analyses: Exp. 3

#### Response time, confidence, and metacognitive sensitivity differences

Participants were slower to deliver ‘no’ responses compared with ‘yes’ responses (median difference: 68.90 ms, *t*(99) =  − 6.36, *p* < .001 for a *t* test on the log-transformed response times; see Fig. A3). No significant difference in response times was observed for the discrimination task (median difference: 11.28 ms, *t*(98) = 0.11, *p* = .912).

Confidence in detection was generally lower than in discrimination, consistent with lower accuracy in this task (*M*_*D*_ =  − 0.04, 95% CI [−0.06, −0.02], *t*(99) =  − 3.87, *p* < .001). Within detection, confidence in ‘yes’ responses was generally higher than confidence in ‘no’ responses (*M* = 0.09, 95% CI [0.07, 0.11], *t*(99) = 8.19, *p* < .001). No difference in average confidence levels was observed between the two discrimination responses (*M* =  − 0.01, 95% CI [−0.03, 0.01], *t*(99) =  − 0.74, *p* = .460).

Within detection, metacognitive sensitivity was higher for ‘yes’ responses (group difference in area under the response-conditional curve, auROC2: *M* = 0.03, 95% CI [0.01, 0.06], *t*(53) = 2.75, *p* = .008). auROC2s were also marginally different when controlling for Type 1 response and confidence biases (*M* = 0.03, 95% CI [0.00, 0.05], *t*(53) = 1.92, *p* = .061).

#### zROC curves

As expected, detection zROC slopes were significantly shallower than 1 (*M* =  − 0.08, 95% CI [−0.11, −0.05], *t*(99) =  − 5.16, *p* < .001), whereas discrimination zROC slopes were not different from 1 (*M* =  − 0.07, 95% CI [−0.23, 0.09], *t*(91) =  − 0.90, *p* = .368). Furthermore, a first-order SDT model fitted the data significantly better for the detection than for the discrimination task (difference in *R*^2^ for the two tasks: *M* = 0.20, 95% CI [0.17, 0.23], *t*(91) = 12.42, *p* < .001).

#### Confidence–RT correlations

We extracted a Spearman correlation coefficient between confidence and response times separately for the two tasks and four responses. We find a negative correlation in all four cases (discrimination responses: −0.37 and −0.45, detection ‘yes’: −0.30, detection ‘no’: −0.25). This negative correlation was significantly attenuated in detection ‘no’ responses compared with detection ‘yes’ responses (tested with a one-tailed *t* test: *t*(99) =  − 2.03, *p* = .023). The difference in correlation strength between detection ‘no’ responses and discrimination responses was also significant (*t*(99) =  − 5.86, *p* < .001).

#### Reverse correlation analysis of standard trials only

In the following, we repeat the reverse correlation analysis reported for Exp. 3, but here restricted to the subset of “standard” trials where luminance was not increased by 2/255.

##### Discrimination decisions

Discrimination decisions were sensitive to relative evidence during the first 300 ms of the trial (*t*(95) = 13.16, *p* < .001) with no effect of sum evidence (*t*(95) = 0.17, *p* = .867).

##### Discrimination confidence

Decision confidence was sensitive to positive (*t*(99) = 6.21, *p* < .001) and negative (*t*(99) =  − 3.37, *p* = .001), evidence within the first 300 ms of the stimulus. The effect of sum evidence on decision confidence was not significant in this sample (*t*(99) = 1.40, *p* = .165).

##### Detection

Participants’ detection decisions were sensitive to fluctuations in the luminance of the target stimulus, such that ‘yes’ responses were associated with brighter target stimuli (*t*(99) = 10.31, *p* < .001). The luminance of the nontarget stimulus had a negative effect on the probability of responding ‘yes’ in the detection task (*t*(99) =  − 5.35, *p* < .001). Together, detection decisions were sensitive to relative evidence (the difference in luminance between the target and nontarget stimuli; *t*(99) = 11.59, *p* < .001), and to sum evidence (the overall luminance of the display *t*(99) = 4.09, *p* < .001).

Confidence in detection ‘yes’ responses was similarly positively correlated with the luminance of the target stimulus (*t*(99) = 5.62, *p* < .001) and negatively correlated with the luminance of the nontarget stimulus (*t*(99) =  − 3.07, *p* = .003). Sum luminance had a significant positive effect on decision confidence when reporting target presence (‘yes’ responses, *t*(99) = 2.07, *p* = .041).

Finally, confidence in detection ‘no’ responses was not sensitive to random fluctuations in the luminance of the target (*t*(95) =  − 0.67, *p* = .503), and nontarget stimulus (*t*(95) =  − 0.02, *p* = .984), nor to the overall luminance of the display (*t*(95) =  − 0.48, *p* = .631), or the difference in luminance between the two stimuli (*t*(95) =  − 0.47, *p* = .637).

### Additional analyses: Exp. 4

#### Response time, confidence, and metacognitive sensitivity differences

Participants were slower to deliver ‘no’ responses compared with ‘yes’ responses (median difference: 48.03 ms; *t*(101) =  − 4.34, *p* < .001, for a *t* test on the log-transformed response times; see Fig. A4). No significant difference in response times was observed for the discrimination task (median difference: 29.77 ms, *t*(109) = 0.50, *p* = .617).

Confidence in detection was generally lower than in discrimination, consistent with lower accuracy in this task (*ΔM* =  − 0.08, 95% CI [−0.13, −0.02], *t*(208.94) =  − 2.86, *p* = .005). Within detection, confidence in ‘yes’ responses was generally higher than confidence in ‘no’ responses (*M* = 0.07, 95% CI [0.05, 0.10], *t*(101) = 5.97, *p* < .001). Within discrimination, confidence in ‘yes’ responses was overall higher (*M* = 0.03, 95% CI [0.01, 0.05], *t*(109) = 2.48, *p* = .015).

Within detection, metacognitive sensitivity was numerically, but not significantly, higher for ‘yes’ responses (group difference in area under the response-conditional curve, auROC2: *M* = 0.03, 95% CI [−0.01, 0.07], *t*(25) = 1.36, *p* = .185). This was the case also when controlling for Type 1 response and confidence biases (*M* = 0.03, 95% CI [−0.02, 0.07], *t*(25) = 1.25, *p* = .223).

#### zROC curves

As expected, detection zROC slopes were significantly shallower than 1 (*M* =  − 0.13, 95% CI [−0.18, −0.08], *t*(101) =  − 5.16, *p* < .001), whereas discrimination zROC slopes were not different from 1 (*M* = 0.05, 95% CI [−0.17, 0.28], *t*(68) = 0.48, *p* = .630). Furthermore, a first-order SDT model fitted the data significantly better for the detection than for the discrimination task (difference in *R*^2^ for the two tasks: *M* = 0.31, 95% CI [0.26, 0.36], *t*(61) = 12.38, *p* < .001).

#### Confidence–RT correlations

We extracted a Spearman correlation coefficient between confidence and response times separately for the two tasks and four responses. We find a negative correlation in all four cases (discrimination responses: −0.38 and −0.36, detection ‘yes’: −0.37, detection ‘no’: −0.32). This negative correlation was significantly attenuated in detection ‘no’ responses compared with detection ‘yes’ responses (tested with a one-tailed *t* test: *t*(101) =  − 2.35, *p* = .010). The difference in correlation strength between detection ‘no’ responses and discrimination responses was also significant (*t*(207.97) =  − 1.86, *p* = .032).

#### Reverse correlation analysis of standard trials only

In the following, we repeat the reverse correlation analysis reported for Exp. 4, but here restricted to the subset of “standard” trials where overall hue was not shifted toward the target hue.

##### Discrimination decisions

Discrimination decisions were sensitive to relative evidence during the first 300 ms of the trial (*t*(82) = 9.45, *p* < .001) with no effect of sum evidence (*t*(82) = 1.74, *p* = .086).

##### Discrimination confidence

Decision confidence was sensitive to positive (*t*(107) = 5.32, *p* < .001) and negative (*t*(107) =  − 3.85, *p* < .001) evidence within the first 300 ms of the stimulus. The effect of sum evidence on decision confidence was not significant in this sample (*t*(107) = 1.36, *p* = .176).

##### Detection

Participants’ detection decisions were sensitive to fluctuations in the hue of the target stimulus (*t*(101) = 9.51, *p* < .001). The hue of the nontarget stimulus had a negative effect on the probability of responding ‘yes’ in the detection task (*t*(101) =  − 3.13, *p* = .002). Together, detection decisions were sensitive to relative evidence (the difference in hue between the target and nontarget stimuli, *t*(101) = 9.13, *p* < .001), and to sum evidence (the overall evidence in hue-space for the two stimuli together, *t*(101) = 4.92, *p* < .001).

Confidence in detection ‘yes’ responses was similarly positively correlated with the hue of the target stimulus (*t*(98) = 3.35, *p* = .001), but was not correlated with the hue of the nontarget stimulus (*t*(98) =  − 0.57, *p* = .570). Sum evidence in hue space had a marginal positive effect on decision confidence when reporting target presence (‘yes’ responses, *t*(98) = 1.89, *p* = .062).

Finally, confidence in detection ‘no’ responses was negatively sensitive to random fluctuations in the hue of the target (*t*(97) =  − 3.32, *p* = .001), but not the nontarget stimulus (*t*(97) =  − 1.51, *p* = .135). The overall hue of the display also affected confidence in decisions about absence (*t*(97) =  − 3.22, *p* = .002), without an effect for the hue difference between the two stimuli (*t*(97) =  − 1.19, *p* = .239).

### Effects of evidence on decision and confidence: Exps. 2 and 3

We plotted optimal behaviour, as well as participants’ responses and their confidence in correct responses, as a function of perceptual evidence in a two-dimensional representational space. First, for each trial we extracted mean luminance (minus background luminance) in the first 300 ms for the right and left stimuli. These numbers were rounded to the closest integer. For each tuple of such integers, we extracted the posterior probability for stimulus category (Fig. A5, top row), participants’ empirical discrimination and detection decisions (middle row), and participants’ subjective confidence in correct responses (bottom row).

### Correlations between detection decisions and discrimination confidence

If discrimination confidence and detection decisions draw on shared representational resources, one may expect that subjects who report higher levels of confidence in detection will also be more likely to report target presence in detection. This was not the case in any of the four experiments (Exp. 1: *r* =  − .40, 95% CI [−.82,  . 30], *t*(8) =  − 1.25, *p* = .248; Exp. 2: *r* =  − .08, 95% CI [−.27,  . 11], *t*(100) =  − 0.83, *p* = .406; Exp. 3: *r* = .00, 95% CI [−.20,  . 20], *t*(98) =  − 0.01, *p* = .990; Exp. 4: *r* =  − .06, 95% CI [−.26,  . 13], *t*(100) =  − 0.65, *p* = .518).

Similarly, discrimination confidence effects of the evidence boost manipulation in Exps. 3 and 4 were not correlated with detection decision effects of the same participants (Exp. 3: *r* = .06, 95% CI [−.14,  . 25], *t*(98) = 0.57, *p* = .569; Exp. 4: *r* =  − .03, 95% CI [−.22,  . 16], *t*(100) =  − 0.31, *p* = .755).

### Computational models

#### General framework

##### Generative model

Stimuli were represented as two vectors of 12 values each ${\overrightarrow{E}}_l$ and ${\overrightarrow{E}}_r$, corresponding to the two sensory channels (e.g., the right and left stimuli in Exp. 2). In the discrimination task, one sensory channel transmitted pure noise (that is, samples were centered around zero), and one channel had additional signal in it (samples were centered around a nonzero value). The signal channel was chosen randomly for each trial with equal probability. In the detection task, both sensory channels transmitted pure noise.

On top of the presented noise, we added perceptual noise to the stimulus, resulting in a degraded representation of each sensory channel *E*^′^. Importantly, this additional noise affected the agent’s decisions and confidence ratings, but did not affect the stimulus itself such that trial-wise estimates of stimulus energy were unaffected for the reverse correlation analysis. The noise was channel and time specific.

##### Belief update

Agents kept track of three quantities, the log likelihood for signal in the right versus the left channels (*LLR*_*r*_), and the log likelihood for the presence of signal in one of the channels, versus noise in both channels (*LLR*_*p*_). Both values were set to 0 at the beginning of each trial. They were then updated from the second time point and on (we used the first time point as a control, to make sure reverse correlation analysis is not showing any effect of evidence at this time point). Log likelihood ratios were updated according to the following rule, where *i* indexes time point within the trial:

$$LL{R}_{r,i+1}= LL{R}_{r,i}+\mathit{\log}\left({P}_s\left(E^{\prime}_{r,i}\right)\right)+\mathit{\log}\left({P}_n\left(E^{\prime}_{l,i}\right)\right)-\mathit{\log}\left({P}_n\left(E^{\prime}_{r,i}\right)\right)-\mathit{\log}\left({P}_s\left(E^{\prime}_{l,i}\right)\right)$$

and

$$LL{R}_{p,i+1}= LL{R}_{p,i}+\mathit{\log}\left({p}_{right}{P}_s\left(E^{\prime}_{r,i}\right){P}_n\left(E^{\prime}_{l,i}\right)+{p}_{left}{P}_n\left(E^{\prime}_{r,i}\right){P}_s\left(E^{\prime}_{l,i}\right)\right)-\mathit{\log}\left({P}_n\left(E^{\prime}_{r,i}\right)\right)+\mathit{\log}\left({P}_n\left(E^{\prime}_{l,i}\right)\right),$$

where *P*_*s*_ is the true probability density function of *E*^′^ values conditioned on signal being present in the channel, and *P*_*n*_ is the true probability density function of *E*^′^ values conditioned on signal being absent. ${p}_{right}=\frac{e^{LL{R}_{r,i}}}{1+{e}^{LL{R}_{r,i}}}$ is the probability that the signal is in the right channel (based on all previous samples) and *p*_*left*_ = 1 − *p*_*right*_ is the probability that the signal is in the left channel (conditioned on signal presence). Note that subjects are rationally incorporating accurate beliefs about the effect of sensory noise on evidence strength in updating their beliefs about the world state.

##### Decision

In discrimination, agents decided ‘right’ when *LLR*_*r*_ > 0 and ‘left’ otherwise. In detection, agents decided ‘present’ when *LLR*_*p*_ > 0 and ‘absent’ otherwise.

##### Confidence

Confidence was the probability of being correct, given an equal prior over the two world states. This equals $\max\left(\frac{e^{LLR_{r}}}{1+e^{LLR_{r}}},\;1-\frac{e^{LLR_{r}}}{1+e^{LLR_{r}}}\right)$ in discrimination and $\max\left(\frac{e^{LLR_{p}}}{1+e^{LLR_{p}}},\;1-\frac{e^{LLR_{p}}}{1+e^{LLR_{p}}}\right)$ in detection.

#### Vanilla

In the vanilla model, sensory noise was sampled from $\mathcal{N}\left(0,2\right)$.

A python simulation is available in the project’s GitHub.

#### Firing rate

In the firing rate model, sensory samples were sampled from *Pois*(*max*(0, 20*E*)).

A python simulation is available in the project’s GitHub.

#### Random attention

In the random attention noise model, sensory noise was sampled from $\mathcal{N}\left(0,2\right)$. However, subjects had access to only one sensory channel per time point.

At the beginning of each trial, one of the two channels was chosen at random with equal probability to be the preferred channel. Then, at each time point, one of the two channels was selected to be attended, with a 0.95 probability of being the preferred channel. Subjects then used information from this channel only to update their beliefs. For example, upon attending the left channel:

$$LL{R}_{r,i+1}= LL{R}_{r,i}+\mathit{\log}\left({P}_n\left(E^{\prime}_{l,i}\right)\right)-\mathit{\log}\left({P}_s\left(E^{\prime}_{l,i}\right)\right)$$

and

$$LL{R}_{p,i+1}= LL{R}_{p,i}+\mathit{\log}\left({p}_{right}{P}_n\left(E^{\prime}_{l,i}\right)+{p}_{left}{P}_s\left(E^{\prime}_{l,i}\right)\right)-\mathit{\log}\left({P}_n\left(E^{\prime}_{l,i}\right)\right).$$

A python simulation is available in the project’s GitHub.

#### Goal-directed attention

In the goal-directed attention noise model, sensory noise was sampled from $\mathcal{N}\left(0,2\right)$. However, subjects had only access to one sensory channel per time point.

The probability of attending the right channel was set to *S*(*LLR*_*r*_), where $S(x)=\frac{1}{1+{e}^{-5x}}$. This made agents heavily biased to attend the channel that is more likely to include signal.

A python simulation is available in the project’s GitHub.

### Time resolved decision and confidence kernels from model simulations

In the main paper, qualitative model predictions were derived after averaging over time points. Here, Fig. A6, A7, A8, and A9 depict time-resolved decision and confidence kernels. As a sanity check, in these simulations agents only incorporated evidence from the second time point and on, allowing us to verify that evidence in the first time point has zero contribution to decision and confidence kernels.

One noteworthy pattern is that the effect of sum evidence on discrimination confidence in the goal-directed model grows over time within a trial (see Fig. A8C, black curve). This is because in this model, the probability of attending the signal channel increases with every time point, making agents increasingly blind to evidence in the nonsignal channel, and increasingly sensitive to evidence in the signal channel. To quantify this effect, we contrasted the time points at which the relative- and sum-evidence confidence kernels peaked. Indeed, in the goal-directed attention model, the sum-evidence kernel peaked significantly later (*t*(199) = 2.52, *p* = .013). This was not the case in any of the other models (vanilla: *t*(199) =  − 1.28, *p* = .203; firing rate: *t*(99) = 1.01, *p* = .315; random attention: *t*(199) = 0.34, *p* = .731).

The same effect was not present in empirical kernels (Exp. 1: *t*(9) = 0.15, *p* = .886; Exp. 2: *t*(100) = 0.36, *p* = .721; Exp. 3: *t*(99) =  − 0.73, *p* = .467; Exp. 4: *t*(108) =  − 1.81, *p* = .073). This null result is difficult to interpret, given that the effect was not very strong in the simulation, and that its magnitude is sensitive to the steepness of the sigmoid function. We therefore do not interpret it further.

### Pseudodiscrimination analysis

In our preregistration document (https://osf.io/d3vkm/), we specified our plan for *pseudodiscrimination analysis*, where we analyze detection ‘signal’ trials as if they were discrimination trials:

In this analysis, we will assume that in the majority of ‘different’ trials, when participants responded ‘yes’ they correctly identified the brighter set. For example, a detection trial in which the brighter set was presented on the right and in which the participant responded ‘yes’ will be treated as a discrimination trial in which the participant responded ‘right’. Conversely, a trial in which the brighter set was presented on the right and in which the participant responded ‘no’ will be treated as a discrimination trial in which the participant responded ‘left’. These hypothetical responses will then be submitted to the same reverse correlation analysis described in the previous section confidence kernels.

We subsequently realized that a much simpler approach is to contrast ‘yes’ and ‘no’ responses for the true and opposite direction of motion (or flickering stimuli) in signal trials. This alternative approach does not entail treating ‘no’ responses as the successful detection of a wrong signal. The results of this analysis mostly agree with the preregistered pseudodiscrimination analysis. For completeness, we include the preregistered pseudodiscrimination analysis for both experiments here.

#### Exp. 1

Pseudodiscrimination decision kernels were highly similar to discrimination decision kernels (see Fig. A10). Here also, motion energy during the first 300 ms of the stimulus had a significant effect on decision (*t*(9) = 4.18, *p* = .002), and on decision confidence (*t*(9) = 3.26, *p* = .010). However, unlike in discrimination, where motion energy in the chosen direction influenced decision confidence more than motion energy in the unchosen direction, no such positive evidence bias was observed for detection responses (*t*(9) = 0.20, *p* = .849).

While motion energy during the first 300 ms of the trial significantly affected confidence in ‘yes’ responses (*t*(9) = 5.52, *p* < .001), it had no significant effect on confidence in ‘no’ responses (*t*(9) =  − 0.09, *p* = .932). However, given that the pseudodiscrimination analysis was performed on signal trials only, confidence kernels for ‘no’ responses were based on fewer trials than confidence kernels for ‘yes’ responses, such that the absence of a significant effect in ‘no’ responses may reflect insufficient statistical power to detect one.

#### Exp. 2

Similar to decision kernels in Exp. 2, random fluctuations in luminance during the first 300 ms of the stimulus had a significant effect on decision (*t*(101) = 6.68, *p* < .001; see Fig. A11). However, in Exp. 2, this analysis revealed no effect of luminance on decision confidence (*t*(99) = 1.36, *p* = .178), and no positive evidence bias in confidence judgments (*t*(99) =  − 0.66, *p* = .512).

## Footnotes

1 We reused the original MATLAB code that was used for Exp. 1 in Zylberberg et al. (2012), kindly shared by Ariel Zylberberg.

2 We thank an anonymous reviewer for bringing this issue to our attention.
